# Purification and proteomics of pathogen-modified vacuoles and membranes

**DOI:** 10.3389/fcimb.2015.00048

**Published:** 2015-06-02

**Authors:** Jo-Ana Herweg, Nicole Hansmeier, Andreas Otto, Anna C. Geffken, Prema Subbarayal, Bhupesh K. Prusty, Dörte Becher, Michael Hensel, Ulrich E. Schaible, Thomas Rudel, Hubert Hilbi

**Affiliations:** ^1^Chair of Microbiology, Biocenter, University of WürzburgWürzburg, Germany; ^2^Division of Microbiology, University of OsnabrückOsnabrück, Germany; ^3^Institute of Microbiology, Ernst-Moritz-Arndt University GreifswaldGreifswald, Germany; ^4^Priority Area Infections, Cellular Microbiology, Research Center Borstel, Leibniz Center for Medicine and BiosciencesBorstel, Germany; ^5^Department of Medicine, Max von Pettenkofer Institute, Ludwig-Maximilians University MunichMunich, Germany; ^6^Department of Medicine, Institute of Medical Microbiology, University of ZürichZürich, Switzerland

**Keywords:** *Chlamydia*, host-pathogen interactions, immuno-magnetic purification, *Legionella*, *Mycobacterium*, *Salmonella*, *Simkania*, pathogen vacuole

## Abstract

Certain pathogenic bacteria adopt an intracellular lifestyle and proliferate in eukaryotic host cells. The intracellular niche protects the bacteria from cellular and humoral components of the mammalian immune system, and at the same time, allows the bacteria to gain access to otherwise restricted nutrient sources. Yet, intracellular protection and access to nutrients comes with a price, i.e., the bacteria need to overcome cell-autonomous defense mechanisms, such as the bactericidal endocytic pathway. While a few bacteria rupture the early phagosome and escape into the host cytoplasm, most intracellular pathogens form a distinct, degradation-resistant and replication-permissive membranous compartment. Intracellular bacteria that form unique pathogen vacuoles include *Legionella*, *Mycobacterium*, *Chlamydia*, *Simkania*, and *Salmonella* species. In order to understand the formation of these pathogen niches on a global scale and in a comprehensive and quantitative manner, an inventory of compartment-associated host factors is required. To this end, the intact pathogen compartments need to be isolated, purified and biochemically characterized. Here, we review recent progress on the isolation and purification of pathogen-modified vacuoles and membranes, as well as their proteomic characterization by mass spectrometry and different validation approaches. These studies provide the basis for further investigations on the specific mechanisms of pathogen-driven compartment formation.

## Introduction

### Proteomics of pathogen-host interactions

Mass spectrometry (MS)–based proteomics is a powerful technology, allowing the identification and quantification of hundreds of proteins from a single sample (Aebersold and Mann, 2003; Otto et al., 2014). This technique has been employed successfully in a diverse range of areas, including the impact of environmental stressors (Maass et al., 2014; Wenzel et al., 2014), conditions of health and disease (Hofmann et al., 2010; Hansmeier et al., 2012; Kopecka et al., 2015; Lassek et al., 2015) or determinants of bacterial and eukaryotic physiology (Cravatt et al., 2007; Trost et al., 2009; Chao et al., 2010; Picotti et al., 2013; Kohlmann et al., 2014). Over the last years, pathogen-host-interactions were increasingly addressed by proteome studies (Becker et al., 2006; Mattow et al., 2006; Rogers and Foster, 2008; Urwyler et al., 2009; Li et al., 2010; Ansong et al., 2013; Claudi et al., 2014; Hoffmann et al., 2014b), summarized in reviews by Bumann (2010), Hartlova et al. (2011), Schmidt and Völker (2011) and Steinert (2011).

A major challenge of studies in the field of pathogen-host interaction is the limited material available from infection settings. In addition, bacterial proteins are vastly outnumbered by host proteins that may not be involved in the infection process. Hence, “-omics” studies are dealing with at least two different organisms combined in a single analytical setting. Consequently, the difficulty lies in targeting either host proteins or pathogen virulence factors individually or both at the same time in comparative studies (Hartlova et al., 2011) with potentially very asymmetric ratios of protein abundance (Schmidt and Völker, 2011). This problem can only be met with methods that are sensitive and provide a wide dynamic range.

An even greater challenge is the targeted analysis of pathogen-containing host compartments. These fragile organelle-like microenvironments represent the direct contact region between the pathogens and their hosts. However, attempts to purify these compartments using classical organelle enrichment techniques were not very successful. Recently, combinations of classical and improved enrichment and pre-fractionation strategies resolved this problem (see chapters below) and enabled for the first time proteomics surveys of those delicate intracellular compartments.

### Gel-based proteomics

**Figure 1:**
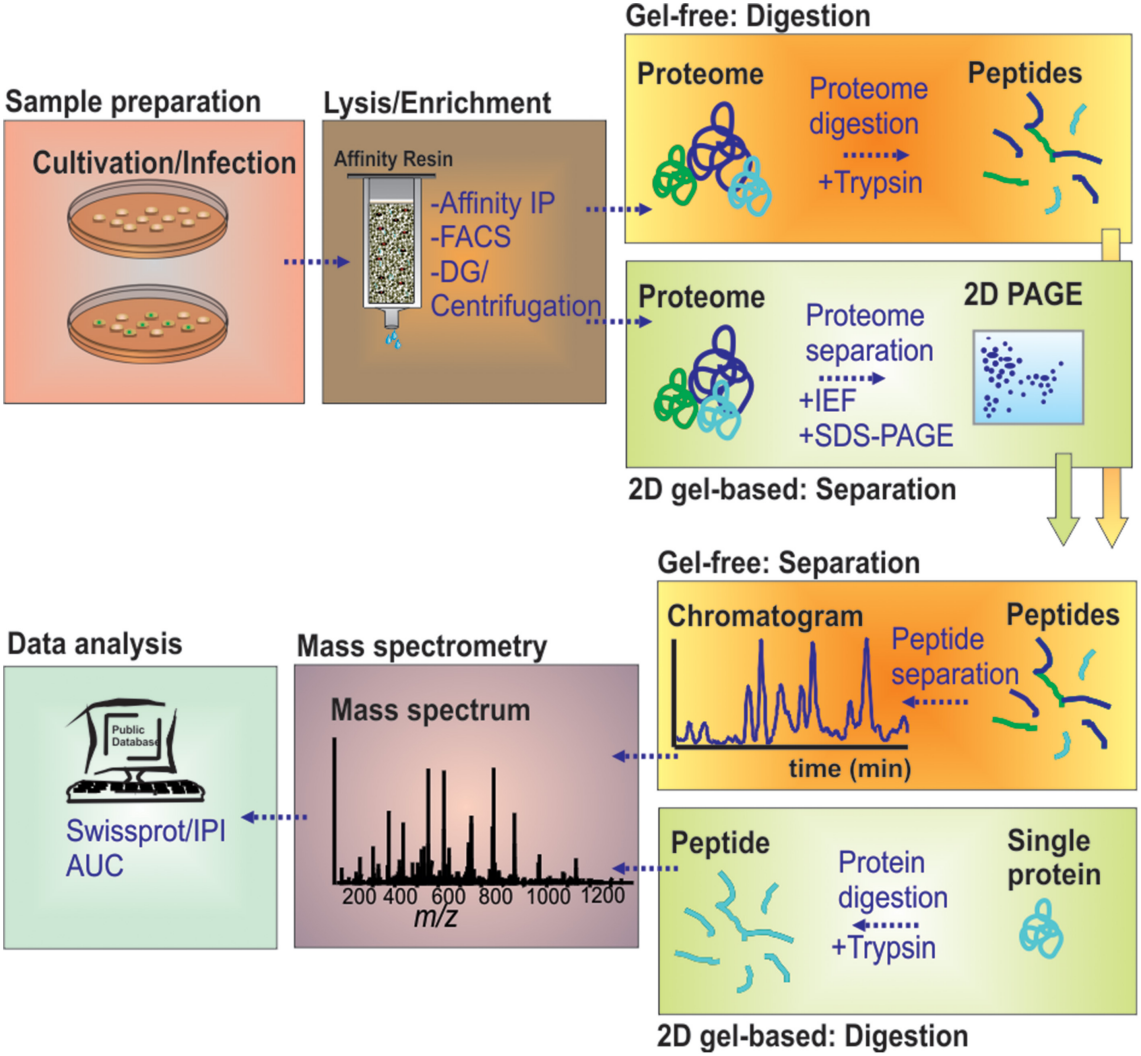
**MS-based workflow of two basic proteomic approaches (gel-based vs. gel-free) to resolve the proteome of pathogen-containing host compartments**. Infected cells were harvested and lysed by chemical or mechanical treatment. Pathogen-containing host compartments were enriched by combinations of density gradient (DG) centrifugation, affinity immuno-precipitation (IP) and fluorescence-activated cell sorting (FACS). Isolated host compartments were then either first enzymatically digested (gel-free), followed by LC-based peptide separation, or first separated by 2D-PAGE followed by individual protein digests, before MS analyses. The data are processed to determine and/or quantify the peptide amino acid sequence through database searches.

The call for global overviews of infection processes on protein level has introduced classical 2D gel-based proteomics early to the field, and it has still kept its place for targeting soluble proteomes (Figure 1). Starting with only a limited number of proteins detected (Desjardins et al., 1994), the analytical depth of global proteome studies has increased markedly by technical developments in 2D gel-based proteomics (Shevchuk et al., 2009). Classical gel-based (i.e., “*top-down*”) proteomics uses two physicochemical properties, the p*I* and the molecular weight, to orthogonally resolve soluble proteins. This is achieved by isoelectric focusing (IEF) followed by SDS-PAGE leading to highly resolved 2D gels. Staining of the protein spots and differential comparison of gel images then allows for a relative quantification across different proteome samples based on protein abundances.

Following spots detection, resolved proteins are identified by state-of-the-art MS techniques, such as matrix-assisted laser desorption ionization time of flight (MALDI-TOF). It is possible to resolve more than a thousand protein spots with up to 700 different proteins by 2D gel-based proteomics. With recent improvements in the second dimension, an increase of detected protein by 25% was achieved (Moche et al., 2013). Despite these improvements, gel-based proteomics suffers from detection limitations due to protein hydrophobicity, abundance and extreme physicochemical properties.

### Gel-free proteomics

The limitations of gel-based proteomics have led to the introduction of liquid chromatography (LC)-MS-based, gel-free proteomics techniques. This has revolutionized the field, in terms of sensitivity and versatility, e.g., allowing to also target membrane proteomes in large scale studies (Aebersold and Mann, 2003; Cravatt et al., 2007; Trost et al., 2009). Gel-free proteomics changed the focus of the analytical setting from the proteins toward proteolytic peptides generated from the samples under investigation (Duncan et al., 2010). In “*bottom-up*” proteomics, protein samples are first digested by specific proteases, mostly trypsin, and the resulting very complex digests are subjected to LC. The peptides are typically loaded on reversed phase columns, desalted and then eluted according to their hydrophobicity by a binary gradient of water/organic solvent. On eluting from the LC-column, the peptides are ionized by electrospray ionization, and mass/charge ratios are determined by MS. The peptides are identified by stochastically sampling the fragment pool, allowing for an unambiguous identification using database searching tools and available sequence databases (Figure 1). This approach is entirely MS-centered, and thus, technology development increasingly determined the sensitivity attained, the protein groups amenable for analyses (membrane proteins, small proteins, basic proteins) and the sheer number or proteins identified.

In contrast to gel-based proteomics, which is regarded as being inherently quantitative, MS-based proteomics approaches are lacking this feature. For differential comparisons most semi-quantitative workflows either depend on the introduction of isotope labels at different stages of sample preparation, both *in vivo* or *in vitro* (metabolic labeling by stable isotope labeling or chemical labeling), or they are label-free and based on for instance “spectral counting” or “area under the curve” determination on the MS1 level (Zybailov et al., 2006; Neilson et al., 2011). Arguably, any *in vivo* stable isotope labeling technique (e.g., SILAC, ^15^N labeling) is superior to other gel-free quantification techniques, but this requires a suitable and redundant biological system that makes labeling at the metabolic level possible (Porteus et al., 2011). However, for the investigation of infection processes, the introduction of stable isotopes by metabolic labeling is challenging; for instance, the simultaneous labeling of host and pathogen is often not possible, thus limiting this approach so far to only defined analytical settings in early stages of infection (Schmidt and Völker, 2011).

A striking feature of gel-free proteomics approaches is the superb sensitivity of high resolution and accurate mass MS. Using this technological advances, it has become possible to investigate host compartments for the presence and composition of host and pathogen proteins, and to elucidate the dynamics of their interactions, as well as to explore post-translational modifications relating to the infection process (Bruckert and Abu Kwaik, 2014). This will be an intense research field in infection biology in the upcoming years.

### Subcellular pathogen compartments

As a pathogen is residing in a specific compartment in the host, the first step for most proteome studies in this field comprises selective enrichment of pathogen-containing vacuoles (PCVs) or internalized bacteria. This may be achieved by subcellular/organellar fractionation based on physicochemical properties (Howe and Heinzen, 2008; He et al., 2012; Cheng et al., 2014), by immuno-affinity purification (Urwyler et al., 2010; Hoffmann et al., 2013; Vorwerk et al., 2015), or through single cell FACS enrichment by sorting internalized bacteria and lysed host cells/organelles (Becker et al., 2006; Pförtner et al., 2013; Surmann et al., 2014). These approaches greatly reduce sample complexity in proteomics approaches and increase the specificity and the quality of conclusions that can be drawn from the data.

For all steps performed during sample preparation, it is crucial to verify the specificity of the chosen approach in order to minimize artifacts caused by co-purified “contaminant” organelles, which are meaningless for the biological setting under investigation (Rogers and Foster, 2007; Shevchuk et al., 2009; Hoffmann et al., 2014a; Vorwerk et al., 2015). Following this enrichment/purification step, any temporal or spatial rearrangement of proteins can be queried by proteomic analytical techniques relying on the aforementioned quantitative or at least semi-quantitative methods (Otto et al., 2014).

Here, we review recent progress on the isolation and purification of pathogen-modified vacuoles and membranes from host cells infected with obligate or facultative intracellular bacteria. To this end, we focus on and compare membrane compartments modified by *Legionella*, *Chlamydia*, *Simkania*, or *Salmonella* spp., respectively, or phagosomes containing beads coated with distinct cell wall lipids purified from *Mycobacterium tuberculosis*. The proteomic characterization of these unique pathogen niches by MS represents a comprehensive approach toward a detailed understanding of these compartments and provides the basis for further investigations on the specific mechanisms of pathogen-driven compartment formation and direct or indirect interactions of pathogen and host factors.

## Intracellular vacuolar pathogens

### Legionella pneumophila

**Figure 2:**
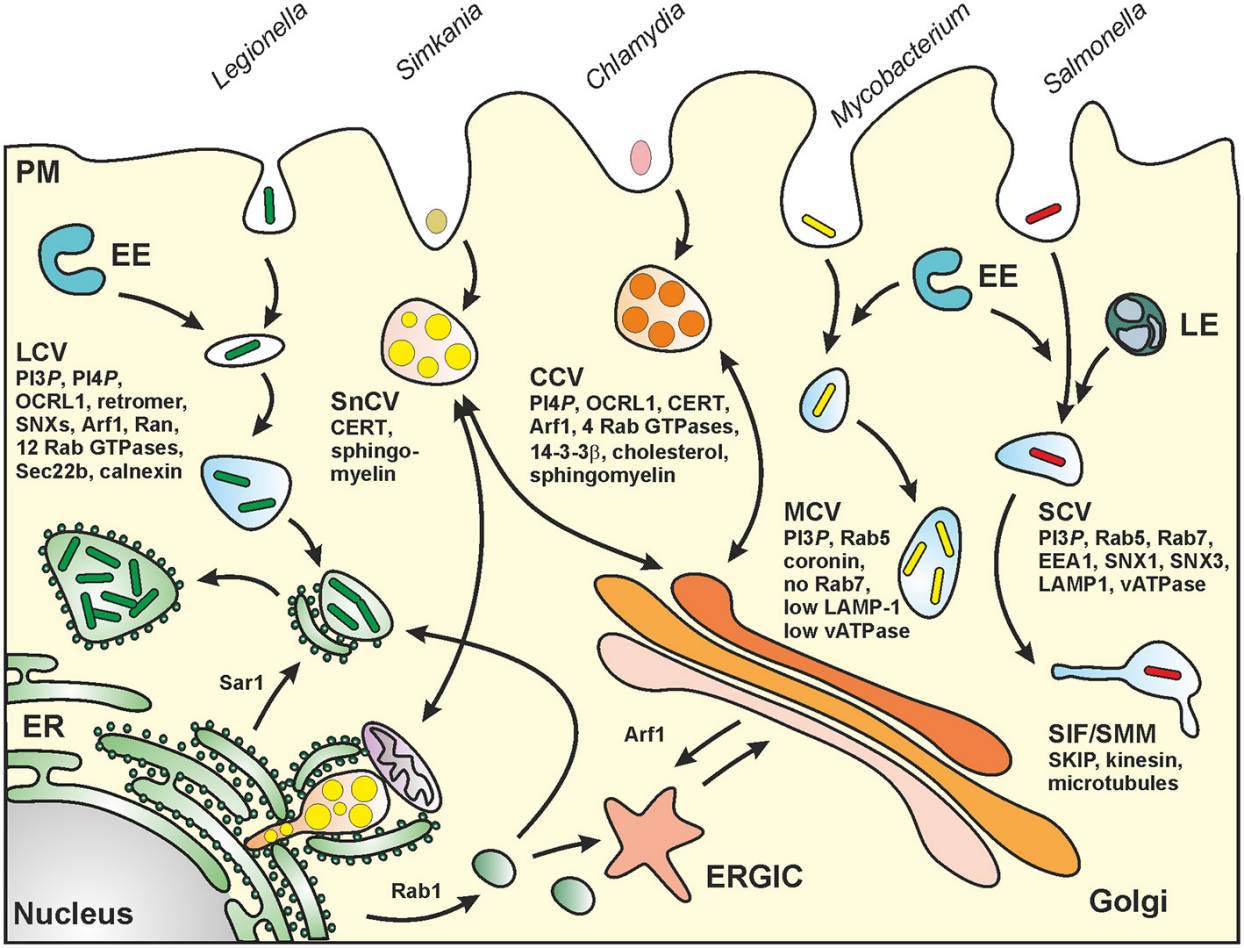
**Biogenesis of pathogen vacuoles**. *Legionella*, *Mycobacterium*, *Chlamydia*, *Simkania*, and *Salmonella* spp. form distinct pathogen-containing vacuoles. Abbreviations: CCV, *Chlamydia*-containing vacuole; CERT, ceramide transfer protein; ER, endoplasmic reticulum; ERGIC, ER-Golgi intermediate compartment; EE, early (sorting) endosomes; LE, late endosomes; LCV, *Legionella*-containing vacuole; MCV, *Mycobacterium*-containing vacuole; PM, plasma membrane; SCV, *Salmonella*-containing vacuole; SIF, *Salmonella*-induced filament; SMM, *Salmonella*-modified membranes; SKIP, SifA and kinesin interacting protein; SnCV, *Simkania*-containing vacuole. LCVs intersect with the secretory pathway between ER exit sites and the *cis*-Golgi network and also interact with the retrograde recycling pathway. MCVs represent maturation-stalled endosomal compartments. *Chlamydia* “elementary bodies” (EBs) form between the TGN and the PM a membrane-bound “inclusion” (CCV), which communicates with the Golgi apparatus and wherein EBs differentiate into “reticulate bodies” (RBs). SnCVs also interact with the Golgi as well as with perinuclear ER and mitochondria. SCVs extensively interact with lysosomes and eventually localize to the peri-Golgi region. For details see text.

*Legionella* spp. are ubiquitous environmental bacteria that upon inhalation cause a severe pneumonia named “Legionnaires' disease” (Newton et al., 2010; Hilbi et al., 2011a). The opportunistic pathogen colonizes a variety of niches in the environment; yet, a peculiar trait of the bacteria is their amoebae-resistance (Hoffmann et al., 2014b). By employing an apparently conserved mechanism, the Gram-negative bacteria replicate intracellularly in free-living protozoa as well as in mammalian macrophages within a unique compartment, the “*Legionella*-containing vacuole” (LCV) (Figure 2). LCVs restrict the fusion with lysosomes and do not acidify, but extensively communicate with the endosomal, secretory and retrograde vesicle trafficking pathways and finally coalesce with the endoplasmic reticulum (ER) (Isberg et al., 2009; Hilbi and Haas, 2012).

Intracellular replication and LCV formation of *Legionella pneumophila* (*Lpn*) requires a bacterial type IV secretion system (T4SS) called Icm/Dot (intracellular multiplication/defective organelle trafficking), which transports “effector” proteins into host cells, where they subvert host vesicle trafficking and signal transduction pathways (Nagai and Kubori, 2011). For *Lpn*, approximately 300 different Icm/Dot substrates have been experimentally validated (Hubber and Roy, 2010; Zhu et al., 2011; Lifshitz et al., 2013), some of which subvert small GTPases of the Arf, Rab, or Ran family (Hubber and Roy, 2010; Itzen and Goody, 2011; Rothmeier et al., 2013; Sherwood and Roy, 2013; Simon et al., 2014), the vacuolar H^+^-ATPase (Xu et al., 2010), the autophagy machinery (Choy et al., 2012), the retromer complex (Finsel et al., 2013b) or phosphoinositide (PI) lipids (Weber et al., 2009; Hilbi et al., 2011b; Haneburger and Hilbi, 2013). E.g., several *Lpn* effectors anchor to the LCV membrane by specifically binding to the PI lipid phosphatidylinositol 4-phosphate (PtdIns(4)*P*) present on the pathogen vacuole together with the phosphatidylinositol 5-phosphatase OCRL1 (Weber et al., 2006, 2014; Ragaz et al., 2008; Brombacher et al., 2009). Thus, *Lpn* modulates in a sophisticated and very specific manner a plethora of host processes to custom-tailor its intracellular replicative niche.

### Mycobacterium tuberculosis

*Mycobacterium tuberculosis* (*Mtb*), the agent of human tuberculosis (TB), is a facultative intracellular pathogen in phagocytes. Upon inhalation, *Mtb* is engulfed by alveolar macrophages (MO) as first line of defense against inhaled pathogens and initiator of host responses to infection. Subsequently, alveolar MO and lung epithelial cells release chemokines including interleukin (IL)-8, thereby amplifying local inflammation by attracting monocyte-derived MO and polymorphonuclear neutrophils (PMN). These cells take their share in engulfing *Mtb*, as do tissue resident dendritic cells (DC), which deliver antigenic cargo to the draining lymph nodes in order to prime specific T cells. *Mtb* induces the release of pro-inflammatory cytokines by MO and DC, which bias T cell immunity toward Th_1_ cell responses, as characterized by generation of the pro-inflammatory and MO-activating cytokines, interferon gamma (IFN-γ) and tumor necrosis factor alpha (TNF-α) (O'Garra et al., 2013). As prime host cells, the resting MO allows intracellular survival and proliferation of *Mtb*, whereas immune activated MO, PMN or DC are not permissive for pathogen growth (Corleis et al., 2012; Weiss and Schaible, 2015). However, despite these early host defense measures, *Mtb* is able to establish infection, which can be contained in more than 90% of all cases at a latent stage but bears the risk of reactivation and development of active TB later in life (Koul et al., 2011).

As *Mtb* fails to actively enter host cells, the bacteria require phagocytosis to reach their intracellular niche, which is facilitated by an array of germ-line encoded innate host cell receptors including C-type lectins such as the macrophage mannose receptor (MMR), MCL and DC-SIGN. Upon opsonization by complement or specific antibodies, CR3 and FcR are also involved in *Mtb* uptake. Simultaneous triggering of Toll-like receptors (TLR-1/2 heterodimers, TLR-9) and other pattern recognition receptors such as Mincle and Dectin-2 perpetuate and enhance inflammatory responses, which in part can limit mycobacterial growth (Tanne and Neyrolles, 2011). MO activation by IFN-γ and TNF-α promotes expression of genes encoding anti-microbial peptides, nitric oxide synthase (NOS2) and LRG47, which directly attack the mycobacteria or mediate microbicidal effector mechanisms, including generation of toxic nitric oxide and oxygen intermediates, host cell apoptosis and autophagy, respectively, and subsequent control of *Mtb* (Weiss and Schaible, 2015).

Upon phagocytosis, virulent *Mtb* converts the newly formed phagosome into a replication-permissive niche, the *Mycobacterium*-containing vacuole (MCV; Figure 2), thereby exploiting the host cell's endosomal system and metabolism to its own benefit and, at the same time, escaping humoral host defense mechanisms such as specific antibodies and complement (Russell, 2011; Weiss and Schaible, 2015). *Mtb* arrests phagosome maturation at an early endosomal stage, as characterized by an almost neutral pH of 6.3, due to limited numbers of the proton-pumping vacuolar ATPase and low concentrations of LAMP-1 and active lysosomal hydrolases (de Chastellier, 2009; Russell, 2011). Furthermore, the *Mtb* phagosome carries the actin-binding coat protein, coronin 1, and acquires iron-saturated holotransferrin (TF), which is delivered through the TF receptor. One benefit of residing in a non-maturing early endosomal phagosome is probably that *Mtb* can access the host cell's iron import pathway to satisfy the pathogen's need for iron (Weiss and Schaible, 2015). Nevertheless, virulent *Mtb* can resist lysosomal conditions, though optimal replication may be hindered in the hostile low pH environment in the phago-lysosome (Ehrt et al., 2015). It has also been reported that virulent *Mtb* can disrupt the phagosomal membrane and escape into the cytoplasm (van der Wel et al., 2007; Simeone et al., 2012), which adds an additional strategy to the virulence properties of this successful and well host-adapted pathogen.

*Mtb* produces several secreted proteins interfering with phagosome maturation. These include Ndk, PtpA and PE_PGRS30. Ndk dephosphorylates and inhibits recruitment of Rab7-GTP and Rab5-GTP to phagosomes (Forrellad et al., 2013). PtpA dephosphorylates VPS33B, a host protein involved in regulation of membrane fusion, and binds to the vacuolar H^+^-ATPase thereby interfering with luminal acidification. The role of PE_PGRS30 is not known although its deletion drives mutant *Mtb* into phago-lysosomes (Forrellad et al., 2013). Recently, the secreted acid phosphatase SapM was found to dephosphorylate phosphatidyl-inositol 3-phosphate (PtdIns3*P*) located at the cytoplasmic sheet of the phagosomal membrane and essential for progression of phagosome biogenesis (Vergne et al., 2005).

Components of the complex mycobacterial cell wall have also been identified as virulence factors, which can deviate phagosome maturation. Examples are mannose-capped lipoarabinomannan (ManLAM), phosphatidyl-inositol mannosides (PIMs) and, most importantly, the cord factor, trehalose-6,6-dimycolate (TDM) (Indrigo et al., 2002; Fratti et al., 2003; Vergne et al., 2003; Kang et al., 2005; Axelrod et al., 2008).

### *Chlamydia* and *simkania* spp.

*Chlamydia* spp. are a major cause of sexually transmitted, pulmonary and ocular diseases in humans worldwide. Recently, other chlamydia-like organisms like *Simkania negevensis* (*Sn*) and *Waddlia chondrophila* (*Wc*) were described to have pathogenic properties as well (Corsaro and Greub, 2006; Baud et al., 2015). Members of the order Chlamydiales are characterized by four particular features: (1) obligate intracellular lifestyle, (2) Gram-negative-related cell wall, (3) coccoid morphology and (4) bi-phasic developmental cycle.

Infection of the eyes by *Chlamydia trachomatis* (*Ctr*) serotypes A-C might lead to chronic conjunctivitis (trachoma) resulting in preventable blindness if untreated, whereas infection of the urogenital tract by serotypes D-L can cause prostatitis, pelvic inflammatory disease and an increased risk of ectopic pregnancy or infertility in women. *Chlamydophila pneumoniae* (*Cpn*) and *Chlamydophila psittaci* (*Cps*) infect the upper respiratory tract and represent community-acquired or animal-transmitted pathogens. Acute infections cause pneumonia or chronic bronchitis and could lead to chronic asthma (Hahn and McDonald, 1998; Harkinezhad et al., 2009). In contrast to *Ctr*, *Cpn* and *Cps* can infect animals like koalas, birds, bovines, musquashs and snails.

*Sn* has a broad host spectrum besides humans and can grow in amoebae and in several human or simian epithelial cell lines or macrophages (Kahane et al., 2007) as well as in insect cells (Sixt et al., 2012). Infection with *Sn* has been connected to upper respiratory tract infections in humans, and manifests as community acquired pneumonia (CAP), bronchiolitis or chronic obstructive lung diseases (Lieberman et al., 2002), and to granuloma formation in reptiles (Soldati et al., 2004). Infections of the lower respiratory tract caused by *Ctr*, *Sn*, and *Wc* have been connected to miscarriages and premature births (Greenberg et al., 2003; Baud et al., 2015).

The life cycles of *Sn* and *Ctr* are similar and consist of two developmental stages. Infection is initiated by endocytosis of so-called elementary bodies (EB). Inside the membrane-bound endosomal compartment, EB differentiate into metabolic active reticulate bodies (RB) that replicate by binary fission in this *Chlamydia*-specific compartment called inclusion or *Chlamydia*-containing vacuole (CCV; Figure 2). Finally, RB re-differentiate into EB that are released by cell lysis or extrusion and start a new infection cycle in surrounding cells (for a review see Bastidas et al., 2013).

The *Ctr* surface proteins OmcB and MOMP mediate adherence to surface receptors of eukaryotic cells that trigger endocytic uptake by host cells (for a review see Mehlitz and Rudel, 2013). This process requires a functional type III secretion system (T3SS) (reviewed by Mueller et al., 2014) to secrete effector proteins, like TARP and CT694, into the host cytosol to modulate the cytoskeleton (for a review see Mehlitz and Rudel, 2013) or lipid transport (reviewed by Elwell and Engel, 2012). The pathogen-containing endosome matures then to CCV by preventing phago-lysosomal fusion and manipulation of host trafficking pathways for nutrient acquisition (Stephens et al., 1998).

Critical mediators of membrane trafficking like Rab1, Rab4, Rab6 and Rab11 (Rzomp et al., 2003; Capmany and Damiani, 2010) are recruited to inclusion membranes, e.g., by direct binding to chlamydial inclusion membrane (Inc) proteins (Rab4-CT229) (Moorhead et al., 2007) or by so far unknown mechanisms (Rab6-BICD1) (Moorhead et al., 2010). These Rab proteins support *Ctr* infectivity and replication (Rejman Lipinski et al., 2009; Capmany and Damiani, 2010). The phosphatidylinositol 5-phosphatase OCRL1 is recruited to the CCV in a Rab-dependent manner to modify the parasitophorous vacuole by production of PtdIns(4)*P* (Moorhead et al., 2010). Additional host factors like Arf1 and PI4KIIα are recruited to provide PtdIns(4)*P* synthesis at the Golgi apparatus. Lipid acquisition (cholesterol, sphingomyelin) is connected to transport of Golgi-derived exocytic vesicles (Carabeo et al., 2003), multivesicular bodies (MVBs) (Beatty, 2006, 2008) and to non-vesicular transport via membrane contact sites to the ER (e.g. CERT-dependent) (Derre et al., 2011; Elwell et al., 2011).

Although the chlamydial inclusion seems to build a single compartment in the cytosol, it interacts with multiple subcellular compartments (Golgi, ER, lipid droplets, mitochondria, and recycling endosomes) to acquire nutrients for successful intracellular replication. *Sn* encodes a functional T3SS as well as T4SS and actively interferes with pro-apoptotic signaling as well as ER stress (Collingro et al., 2011; Mehlitz et al., 2014). In contrast to the CCV, the *Simkania*-containing vacuole (SnCV; Figure 2) represents an inhomogeneous compartment that develops multiple ER-contact sides. Thereby, the vacuole seems to grow along the rough and smooth ER (Mehlitz et al., 2014).

### Salmonella enterica

*Salmonella enterica* (*Sen*) is a food-borne Gram-negative pathogen causing a high number of infectious diseases ranging from localized, self-liming gastroenteritis to systemic, life-threatening typhoid fever. *Salmonella* spp. are invasive, facultative intracellular pathogens residing in a unique membrane-bound compartment, termed *Salmonella*-containing vacuole or SCV (Haraga et al., 2008) (Figure 2). Within the SCV, the pathogen deploys the SPI2-encoded type III secretion system (SPI2-T3SS) to translocate effector proteins that manipulate various host cell functions, including vesicular transport and the organization of the endosomal system (Ibarra and Steele-Mortimer, 2009; Rajashekar and Hensel, 2011; Figueira and Holden, 2012). The SCV is considered a unique pathogen-containing compartment that is derived from the pathway of endosomal maturation. SCV markers include the lysosomal glycoprotein LAMP1 as well as the small GTPase Rab7, and the lumen of SCVs is thought to have a pH around 5.

The ability of *Sen* to survive within eukaryotic host cells is closely linked to systemic pathogenesis. Mutant strains defective in intracellular survival and replication such as SPI2-T3SS-deficient strains are highly attenuated in systemic disease models of infection (Hensel et al., 1998). One dramatic consequence of host manipulation is the induction of complex networks of tubular membrane compartments, such as *Salmonella*-induced filaments (SIF) that are characterized by the presence of late endosomal/lysosomal membrane proteins (Garcia-del Portillo et al., 1993). Recently, further *Salmonella*-induced tubular compartments have been described in addition to SIF, termed *Salmonella*-induced SCAMP3 tubules (SIST) and LAMP1-negative tubules (LNT) (Schroeder et al., 2011). In order to understand the intracellular lifestyle of *Salmonella* in mammalian host cells, the characterization of the specific properties of the SCV is a key issue.

## Isolation and purification of pathogen vacuoles and membranes

### *Legionella*-containing vacuoles

**Figure 3:**
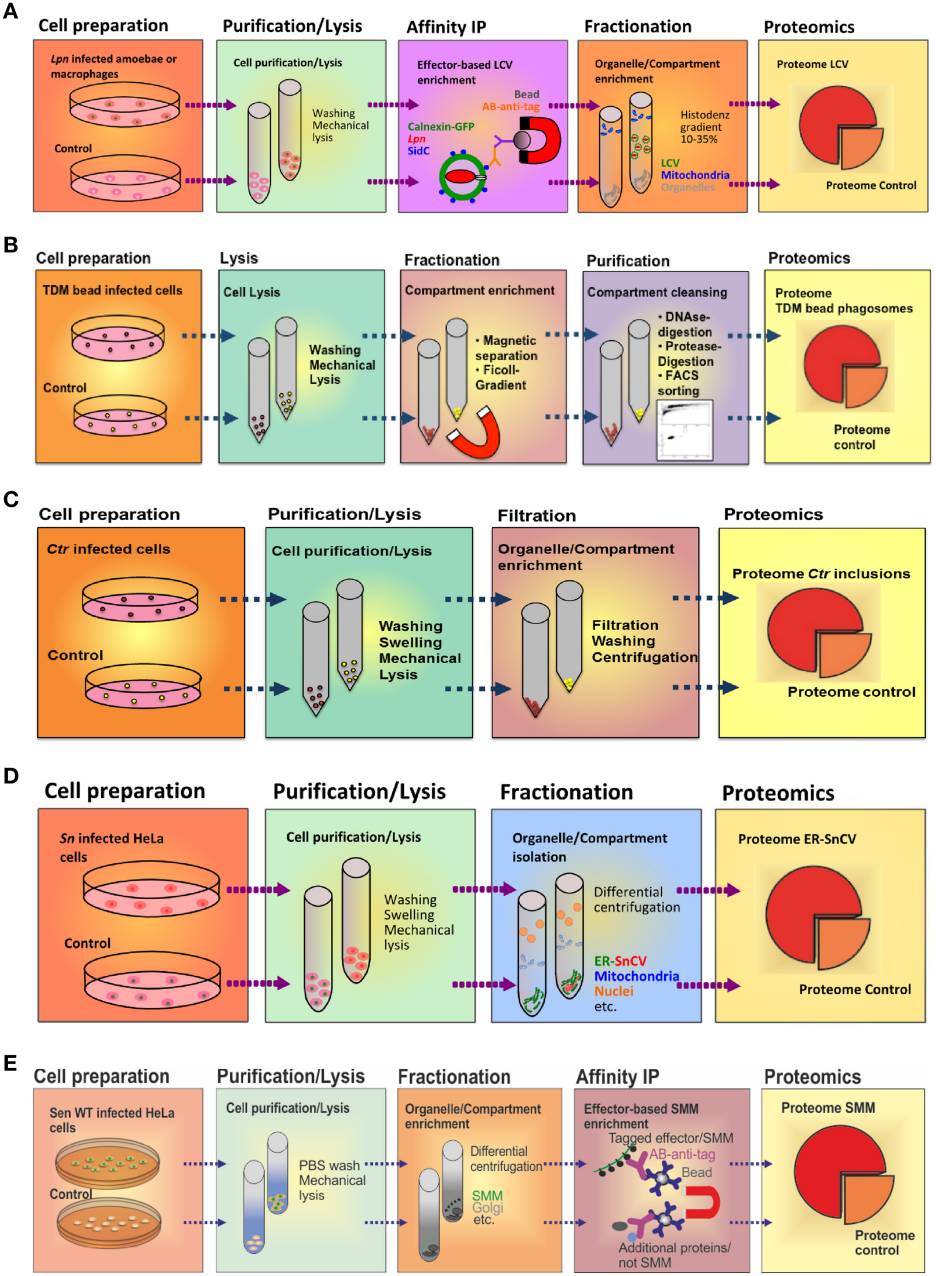
**Purification schemes of pathogen vacuoles and pathogen-modified membranes. (A)** Purification of *Legionella*-containing vacuoles (LCVs) from infected (1 h) *D. discoideum* or RAW 264.7 macrophages by immuno-magnetic separation using an anti-SidC antibody, followed by Histodenz density gradient centrifugation. **(B)** Enrichment of *Mycobacterium* TDM-bead phagosomes by magnetic purification, Ficoll density gradient centrifugation and FACS sorting. RAW 264.7 macrophages exposed to TDM-beads were lysed after 30 min, and phagosomes were isolated (DynaMag magnet), followed by density gradient centrifugation, DNase and gentle protease treatment, and finally by sorting via FACS. Isolation of **(C)** *Chlamydia*-containing vacuoles (CCVs) or **(D)** *Simkania*-containing vacuoles (SnCVs) were separated by differential centrifugation. *Ctr*-infected HeLa cells (24 h) were washed, trypsinised, resuspended in swelling buffer and lysed with a Dounce homogenizer, followed by double filtration. *Sn*-infected HeLa cells (72 h) were scraped, resuspended in swelling buffer, lysed with a Douncer and an ultrasonic bath, followed by sequential centrifugation. **(E)** Enrichment of fractionated *Salmonella*-modified membranes (SMMs) using differential centrifugation followed by immuno-precipitation (IP) with antibodies against the epitope-tagged effector protein SseF. PNF, pre-nuclear fraction; PMF, pre-mitochondrial fraction.

The LCV is a complex pathogen compartment formed by continuous interactions with a variety of host cell organelles and trafficking pathways. To enrich intact LCVs, we sought to exploit the fact that some Icm/Dot T4SS substrates exclusively localize to the pathogen compartment membrane. E.g., the 106 kDa Icm/Dot substrate SidC selectively anchors to the LCV membrane (Luo and Isberg, 2004) and specifically binds to the host cell PI lipid PtdIns(4)*P via* a C-terminal 20 kDa domain termed “P4C” (PtdIns(4)*P*-binding of SidC) (Weber et al., 2006; Ragaz et al., 2008; Brombacher et al., 2009; Dolinsky et al., 2014). Using an affinity-purified polyclonal anti-SidC antibody, we established a straight-forward two-step protocol to isolate intact LCVs from *Lpn*-infected *Dictyostelium discoideum* amoebae (Urwyler et al., 2010; Finsel et al., 2013a) or murine macrophage-like RAW 264.7 cells (Hoffmann et al., 2012, 2013) (Figure 3A). The LCV purification protocol is based on immuno-magnetic separation followed by classical density gradient centrifugation, and it allows monitoring the enrichment of pathogen vacuoles by light microscopy using fluorescently labeled *Lpn* and phagocytes.

To isolate LCVs from phagocytes, *D. discoideum* amoebae producing the LCV/ER marker calnexin-GFP or RAW 264.7 macrophages were infected at a multiplicity of infection (MOI) of 50 with *Lpn* producing the red fluorescent protein DsRed and incubated for 1 h at 25°C (*D. discoideum*) or 37°C (macrophages). The infected phagocytes were washed with SorC buffer (*D. discoideum*) or PBS (macrophages) and scraped in osmo-stabilizing homogenization buffer (Derre and Isberg, 2004). Subsequently, the cells were homogenized using a stainless steel ball homogenizer (8 μm clearance; Isobiotec) and incubated with an anti-SidC antibody, followed by a secondary anti-rabbit antibody coupled to magnetic beads. The LCVs in the homogenate were separated in a magnetic field using a MACS multistand (Miltenyi Biotec) and further purified by Histodenz density gradient centrifugation.

The purified LCVs thus obtained were analyzed by MS, Western blot and immuno-fluorescence microscopy (Hoffmann et al., 2014a). Moreover, host and pathogen LCV factors identified by MS were validated by fluorescence microscopy of intact infected cells, and their functional role for LCV formation was assessed by using defined *Lpn* or *D. discoideum* deletion mutants or by RNA interference in epithelial cells (Rothmeier et al., 2013; Hoffmann et al., 2014a; Simon et al., 2014).

### *Mycobacterium* TDM-beads phagosomes

To characterize the virulence function of purified mycobacterial cell wall lipids in *Mtb* phagosome biogenesis, a simplified glycolipid-coated bead model was employed (Axelrod et al., 2008; Geffken et al., 2015). This approach differs from the protocols described above for analysis of other pathogen-containing vacuoles. The simplified “infection model” using beads coated with TDM was employed to obtain purified phagosomes that are determined by just one mycobacterial virulence factor. This concept allowed to analyze better defined phagosomes in proteomics studies, and to establish a more restricted TDM interactome, some candidates of which were subsequently validated in the *Mtb*-MO infection model (Kolonko et al., 2014).

In short, bovine-serum-albumin (BSA) was covalently linked to magnetic polystyrene beads (Dynabeads, Dynal) via ester-bonds facilitated by the tosyl-activated surface of the beads. The lipid-binding capacity of BSA was employed to coat the beads with purified cell wall glycolipids of *Mtb*. These beads were used to mimic infection of MO to monitor phagosome biogenesis in the presence or absence of *Mtb* glycolipids. After different periods of time, bead phagosomes were analyzed either by microscopy or, upon purification, by Western blot, lysosomal enzyme tests and FACS. Thereby, we identified trehalose 6,6′-dimycolate (TDM), an abundant mycobacterial cell wall glycolipid, as essential *Mtb* virulence factor. TDM efficiently decelerates bead phagosome maturation when coated to the bead surface (Axelrod et al., 2008). Of note, intracellular mycobacteria show increased production of TDM when compared to those grown in broth, indicating a function of TDM in the intracellular phase of *Mtb* (Fischer et al., 2001). Furthermore, using the reductionist lipid-on-bead model, we revealed that the virulence function of TDM, i.e., decelerating phagosome maturation, is abolished in IFN-γ-activated MO by nitric oxide (Axelrod et al., 2008).

Considering TDM an essential virulence determinant of *Mtb*, identification of putative host target proteins is a prerequisite to understand the function of the lipid in inhibiting phagosome maturation. To achieve this task, we used a comparative proteomics approach analyzing purified phagosomes containing either control or TDM-coated beads. The procedure to purify bead phagosomes from macrophages has been described in detail (Geffken et al., 2015) (Figure 3B). Briefly, RAW 264.7 macrophages were “bead-infected” using dynabeads covalently coated with BSA and TDM, incubated for 30 min at 37°C and scraped into ice-cold PBS. The pellets were then resuspended in ice-cold homogenization buffer (HB) and disrupted using a metal Douncer. Lysates were further pushed through a needle, incubated with DNase I and washed in ice-cold PBS using the DynaMag-2 magnet. To further remove cell-debris, samples were digested with trypsin, washed three times with HB and loaded onto a 15% Ficoll-gradient. Subsequently, the samples were diluted in ice-cold PBS, sorted by FACS, and phagosomes were collected by using a magnet. Remaining proteins in supernatants were precipitated using “StrataClean Beads.”

The iron content of the beads was used for quantification of purified bead-phagosomes by determining iron-concentration (Zhu et al., 2012). For quality control of bead-phagosome preparations we used the lysosomal β-galactosidase assay as described previously (Lührmann and Haas, 2000). Pairs of FACS-purified control- or TDM-dynabead-phagosomes and their corresponding StrataClean precipitated supernatants were further processed for LC-MS/MS analysis. To determine the phagosomal proteome, we used GeLCMS as described (Bonn et al., 2014). In this approach, isolated proteins were gel-separated before being subjected to tryptic digestion and RP-LC-MS/MS analyses using an Orbitrap Elite coupled online to an EASY-nLC 1000 (Thermo, Bremen).

### *Chlamydia* and *simkania* inclusions

The knowledge of how *Ctr* can effectively control the signaling pathways and escape from the host cell death pathway depends on the identification and analysis of the host cell factors that interact with the *Ctr* inclusion. To identify these host proteins, a method for the native *Ctr* inclusion isolation was established (Figure 3C). Inclusions were isolated and purified as previously described (Matsumoto, 1981); yet, the protocol included several modifications as outlined below. Since the purification relies on a double filtration procedure, non-infected cells were used as a control to eliminate proteins that pass the filters in absence of inclusions.

HeLa cells were infected with *Ctr* at a MOI of 1 for 24 h. After washing the cells twice with PBS, cells were treated with trypsin and collected. Subsequently, cells were washed three times with PBS, re-suspended in hypotonic swelling buffer and kept on ice for 90 min. Swollen cells were homogenized using a glass Dounce homogenizer, filtered (11 μm nylon filter) and centrifuged. The supernatant was passed through a second glass membrane filter, centrifuged and washed twice. Further washing and centrifugation steps were applied if debris was visible close to the inclusion pellet. The pellet containing the inclusions was finally re-suspended in sucrose-Tris buffer containing 5% BSA and spun down. The purification procedure was monitored by phase contrast, confocal or electron microscopy and immuno-blot analysis. Pure inclusions were enriched and seen abundantly at the final stage of purification.

Through this approach, intact *Ctr* inclusions were obtained. However, six dishes of cells were required to yield sufficient amounts of purified inclusions. Further, around 40% of the inclusions tended to break during purification. Therefore, the infected cells were used for inclusion preparation no longer than 24 h post infection (p.i.), since intact inclusions could hardly be obtained after longer infection periods. The integrity of inclusions was tested by analyzing inclusions containing GFP-producing *Chlamydia* (Wang et al., 2011). Another caveat is that weakly interacting host proteins may detach from the chlamydial inclusion membrane during the purification process, which leads to the loss of candidate CCV host components.

In our previous work, we identified a direct association of *Simkania*-containing vacuoles (SnCVs) with the ER of the host (Mehlitz et al., 2014). It is currently unknown how ER-SnCV interaction sites are established and how they may affect the composition of the ER. Therefore, we established an ER-SnCV membrane purification protocol (Herweg et al., in revision) (Figure 3D). ER membranes of non-infected cells were used as control. Since no suitable targets for IP are known, we performed sub-cellular fractionation instead of immuno-magnetic separation. To this end, HeLa 229 or THP1 cells were seeded in 6-well plates 24 h prior to infection with *Sn*. The infected cells were harvested (3 days p.i.) by scraping. Subsequently, the cells were swelled and lysed with a Dounce homogenizer and ultrasonic bath resulting in the release of the bacteria from SnCVs. ER-SnCV membranes were purified by sequential centrifugation including ultracentrifugation. Since *Sn* is not accessible to genetic modifications, bacteria or SnCVs labeled with fluorescent proteins could not be used to validate the purification process. The loss of intact bacteria as well as the purification of ER-SnCV membranes thus had to be monitored using transmission electron microscopy (TEM). ER-SnCV membranes were isolated as a crude microsomal fraction (CMF) to capture the total ER and SnCV membranes. Since organelles like nuclei and mitochondria were depleted from CMF, contaminations from these organelles may originate from their interaction with the SnCV, such as ER contact sites, mitochondria-associated membranes or the continuous part of the nuclear outer membrane. As a final quality control, immuno-blots were used, which revealed a strong enrichment of ER membrane marker proteins (calnexin, KDEL) and SnCV proteins (anti-*Sn*) in the CMF. ER-SnCV membrane purification was performed with approximately 1 × 10^8^ cells per sample for further LC-MS/MS measurements.

### *Salmonella*-modified membranes

We define the sum of host cell membranes that are modified by activities of intracellular *Sen*, such as SCV, SIF, and SIST membranes as *Salmonella*-modified membranes (SMM). The common feature of SMM is the presence of translocated SPI2-T3SS effector proteins such as SseF, a membrane-integral protein localizing to the pathogen vacuole membrane (Muller et al., 2012). The presence or absence of canonical organelle marker proteins has been analyzed for the SCV in various previous studies. Commonly, light microscopy was used to analyze the presence or absence of such host cell compartment marker proteins on the SCV. However, due to the diffraction limit of light microscopy, these approaches only provide limited insight into the spatial organization of the SCV or the biogenesis of SIT, and thus, alternative methods are required.

A systemic proteomic inventory of the membrane proteomes of SCV and SIT has been hampered by the lack of efficient procedures to enrich SCV and SIT membranes by subcellular fractionation. Such proteomes may provide important clues to the biogenesis of the pathogen-containing compartment, as exemplified for *L. pneumophila* (Urwyler et al., 2009; Hoffmann et al., 2014a). However, the complex tubular arrangement of SMM renders futile the enrichment by classical subcellular fractionation. Recently, Vorwerk et al. revised the fractionation procedure for *Salmonella*-infected HeLa cells and established a novel protocol for the isolation and subsequent proteome analyses of SMM (Vorwerk et al., 2015) (Figure 3E). We used a three-step approach, in which cell purification/lysis is followed by intracellular compartment enrichment and affinity immuno- precipitation (IP).

Since the SPI2-T3SS effector SseF is one of the most abundant *Sen* components of SMM, we used an epitope-tagged SseF version as bait for SMM isolation. For this purpose, we constructed a low-copy expression vector with a C-terminal epitope-tagged *sseF* and its cognate chaperone *sscB* and introduced it in an *sseF*-deficient *Sen* mutant strain to avoid effector overproduction and alteration of the infection process. Expression and co-localization of the epitope-tagged SseF was visually validated to exclude impairments of SIF formation. Infection conditions were optimized for maximum bait expression, and sub-cellular compartments were separated and harvested by differential centrifugation before IP (Vorwerk et al., 2015). With this approach, a total of 500 μg protein was gained from enriched compartment fractions of 7 × 10^7^ infected host cells (MOI 50, harvested at 8 h p.i.), which was then used for IP using 25 μl cross-linked anti-epitope tag labeled magnetic beads. The eluted samples were analyzed for presence of effector proteins by Western blot, before profiling with LC-MS/MS. In contrast to the LCV enrichment protocol described above (Urwyler et al., 2009; Hoffmann et al., 2014a), we enriched intracellular compartments prior to IP of SMM.

## Proteomes of pathogen-containing compartments

### *Legionella*-containing vacuoles

The purified LCVs obtained from *Lpn*-infected *D. discoideum* or RAW 264.7 macrophages by the two-step protocol (immuno-magnetic separation and density gradient centrifugation) were subjected to LC-MS/MS (Hoffmann et al., 2014a). The proteomics analysis revealed more than 670 (amoebae) or 1150 (macrophages) host proteins. A large number of these proteins were implicated in immune responses, signal transduction, membrane dynamics, lipid metabolism, transport processes, or cytoskeleton architecture. A comparison of the *D. discoideum* and macrophage LCV proteomes indicated an overlap of 16 or 28%, respectively, of the total proteins identified on the LCV preparations of the two phagocytes. However, if only proteins strictly conserved among macrophages and *D. discoideum* are compared, e.g. Arf, Sar, and Rab family GTPases involved in membrane dynamics, the percentage of common LCV proteins rises to 50%.

Host factors identified in the LCV proteome included a total of 14 small Rab GTPases on pathogen vacuoles purified from *D. discoideum* or macrophages, of which 11 were validated by fluorescence microscopy (Rab1, Rab2, Rab4, Rab5, Rab7, Rab8, Rab10, Rab11, Rab14, Rab21, and Rab32). Rab9 was discovered only by microscopy, and the LCV localization of Rab6, Rab18 or Rab31 could not be verified (Hoffmann et al., 2014a). Furthermore, many of these Rab proteins were confirmed by fluorescence microscopy to localize on *Lpn* vacuoles harboring wild-type but not Δ*icmT* mutant *L. pneumophila*. In addition to the 12 Rab GTPases, also Arf1, Rap1, Ran, and Rho-related GTPases were identified in the LCV proteome. This abundance of small GTPases corroborates the notion that LCVs communicate with various cellular signaling and vesicle trafficking pathways (Hoffmann et al., 2014a). The depletion of individual GTPases by RNA interference in epithelial cells indicated that Rab GTPases involved in endosomal trafficking (Rab5a, Rab14, and Rab21) restrict intracellular growth of *Lpn*, whereas Rab GTPases (Rab8a, Rab10, and Rab32) implicated in secretory trafficking promote bacterial replication.

Finally, the LCV proteome determined 1 h post infection revealed *Lpn* proteins likely required early during intracellular replication. These include a number of high abundance proteins, such as flagellin, components of the central metabolism and bioenergetics machinery, as well as ribosomal and heat shock proteins. However, also many low abundance proteins were identified, including regulatory elements and virulence factors such as components of the Lsp T2SS or the Icm/Dot T4SS and as many as 60 Icm/Dot-translocated effectors.

The intriguing discovery of the small GTPase Ran and its effector RanBP1 in the LCV proteome (Urwyler et al., 2009), together with the finding that the Icm/Dot substrate LegG1 harbors a putative eukaryotic RCC1 Ran GEF domain (de Felipe et al., 2008; Ninio et al., 2009), led to the characterization of the *Lpn* effector protein as the first bacterial Ran activator (Rothmeier et al., 2013). LegG1 localizes to the LCV membrane, promotes microtubule stabilization and intracellular LCV motility as well as replication of *Lpn*. Moreover, through the stabilization of microtubules, LegG1 antagonizes the Icm/Dot-dependent inhibition of chemotactic and random cell migration (Simon et al., 2014). In summary, the validation and functional analysis of the proteome of purified LCVs demonstrates that the host and pathogen components identified represent a useful inventory of factors that play a role in the complex process of pathogen vacuole formation.

### *Mycobacterium* TDM-beads phagosomes

The identification of putative host cell-derived interaction partners of the *Mtb* virulence-associated cell wall lipid TDM was facilitated by proteomic analysis of purified TDM-bead phagosomes. A hallmark of TDM virulence is its ability to interfere with phago-lysosomal fusion upon uptake by MO. This function is also evident, when TDM alone is tested in a reductionist model of coated beads *in vitro*.

RAW 264.7 MO were infected with TDM-coated beads, lysed after 30 min, and bead-phagosomes were purified (Geffken et al., 2015). The dynabead-phagosome preparation was subjected to LC-MS/MS and analyzed via Sequest and Scaffold 4. In total, 835 proteins were identified in the TDM-bead phagosome preparations, of which 542 localized to intracellular organelles. For instance, 137 or 97 proteins were predicted mitochondrial or cytoskeletal proteins. 379 were membrane-associated and predicted to localize with the plasma membrane (181), ER (97), endosomes (69), and/or Golgi (69), respectively.

Proteins specified with the categories ribosome, extracellular region, Golgi, cytoskeleton, ER, mitochondria, nucleus, and cytoplasm are potential contaminant proteins, because per definition of the gene ontology (GO) terms, these groups do not comprise proteins that localize in intracellular vesicles or their membranes. In contrast, the categories intracellular organelle, organelle part or membrane and organelle membrane can contain, per GO term definition, proteins within organized structure of distinctive morphology and function, occurring within the cell or associated with membranes thereof. These categories may be those containing phagosomal or lysosomal proteins. Therefore, our method to purify control and TDM-bead phagosomes provides pure bead phagosome preparations with limited amounts of contaminant proteins, which when present in high abundances can overwrite signals of proteins of interest in MS analysis.

Importantly, proteins not classified as endo- or phagosomal can still be involved in either direct or indirect interaction with bead-phagosomes. For example, cytoskeleton proteins have been identified prominently in TDM-bead phagosome preparations. Cytoskeleton elements however, are functional in phagocytosis and subsequent events during phagosome biogenesis (Flannagan et al., 2012; Weiss and Schaible, 2015). We recently described the role of WASH-mediated actin recruitment to the mycobacterial phagosome as a prerequisite for inhibition of phagosome maturation and intracellular bacterial growth (Kolonko et al., 2014). Taken together, our improved purification protocol (Geffken et al., 2015), combined with high-resolution MS-based proteomics, revealed candidates putatively involved in TDM-bead phagosome formation, and therefore, most likely play a role for *Mtb* interactions within host MO (Kolonko et al., 2014).

### *Chlamydia* and *simkania* inclusions

Isolated *Ctr* inclusions and ER-SnCV membranes as well as control samples were analyzed by LC-MS/MS via Sorcerer. MS data of four biological replicates of purified non-infected and *Ctr*-infected samples were combined. In total, 2671 proteins were identified, of which 2231 were host proteins and 440 were *Ctr* proteins. About 662 human proteins were reproducibly found in all samples, of which 102 proteins (36 enriched and 66 depleted proteins) were differentially expressed (<0.5 or >2-fold). Protein targets that play a crucial role in the regulation of host cell metabolism and apoptosis were chosen for validation studies, because energy acquisition from the host and inhibiting cell death represent important conditions for successful *Ctr* infections (Subbarayal et al., 2015). The association of these factors with chlamydial inclusion and their regulation during infection was studied by immuno-fluorescence microscopy, immuno-blot analysis and RNA interference.

Analyzing the ER-SnCV LC-MS/MS-data was challenging, since a large number of host cell proteins were identified (Herweg et al., in revision). We combined three biological replicates of *Sn* and non-infected cells in a semi-quantitative manner. Using this approach, we identified 1178 human and 302 *Simkania* proteins in the infected samples. Interestingly, we identified many human proteins linked to membrane-enclosed compartments, including the endomembrane system, envelope and vesicles, as well as approximately 15% ER-localizing proteins. Furthermore, proteins of the nucleus, mitochondria, cytosol and cytoskeleton were also identified; although contaminations from co-purifying intact organelles were excluded based on immuno-blot and TEM validations.

Since the ER represents the origin of protein synthesis, we expected to identify proteins from different cellular compartments. Nevertheless, most host proteins that were not associated with the ER-SnCV should be present in similar amounts in purified *Sn* and ER preparations of non-infected samples, if they are not specifically modified by the bacterial infection. The comparison of *Sn*-infected and non-infected samples helped to identify enriched and depleted host cell factors. Consistent differences among the three replicates were considered to reflect potential regulatory aspects. Further analyses demonstrated an effect of seven main transport pathways on SnCV formation (clathrin, COPI, COPII, ER-to-Golgi transport, endosomes, exocytosis, recycling endosomes). Interestingly, we identified inverse protein regulation regarding the retrograde and anterograde transport systems. The influence of the retrograde transport on SnCV formation was further validated by immuno-fluorescence, immuno-blot, TEM and life cell imaging experiments. In the future, a comprehensive RNAi screen targeting several trafficking routes should confirm our observations. The influence of additional trafficking processes could complete our understanding of how the SnCV is formed. Finally, the proteome data also yielded SnCV-specific and possibly secreted bacterial factors. These will be used as targets for immuno-staining of the SnCV in future experiments.

Using epithelial cells or phagocytes, we did not observe cell-dependent pronounced enrichments or depletions of human proteins in the SnCV compared to ER. It is possible that *Sn* is so well adapted to its niche that major modifications of the ER as an organelle central to cell life are avoided. In *Sn*-infected cells apoptosis or ER-stress is prevented for many days and bacterial growth is quite slow (7–12 days per developmental cycle) (Kahane et al., 2002). While the replication rate is strongly reduced, the cells do not enter a persistent phase and do not appear to be stressed. This supports the previously suggested theory that phagocytes containing *Sn* may act like a Trojan horse that silently spreads and distributes the bacteria inside a human host (Greub and Raoult, 2004).

### *Salmonella*-modified membranes

SMM were enriched by IP from sub-cellular fractionated lysates of infected host cells and subsequently profiled by LC-MS/MS (Vorwerk et al., 2015). Altogether, 552 host proteins were reproducibly identified. As with all IP approaches, we could not exclude that beside SMM components also other proteins may be co-isolated. Therefore, we introduced a negative control for a comparative proteomic approach to eliminate unspecific enrichments. To this end, we used the IP-extracted proteome of a *Sen* strain deficient for SPI2-T3SS-translocation. Two hundred and nine proteins from the initially 552 identified SMM host proteins were also identified in this control, reducing the SMM-specific proteome to 243 unique host proteins. Thus, we minimized the number of false-positive identifications. However, it may also lead to an increase of false negatives. Nonetheless, we generated a list of priority SMM targets, of which 11 proteins were found to be part of or interact with SMM using orthogonal methods such as immuno-staining and live cell imaging (Vorwerk et al., 2015).

Analyses of the SMM components revealed that proteins from different cellular origins were included in the pathogen-modified membranes. In line with previous observations, a large number of cytoskeletal, endo- and lysosomal, as well as Golgi and vesicle transport-related proteins were identified. Moreover, many proteins previously not considered as present on SCV and SIF membranes were observed. In addition to late endosomal compartments already known to interact with the SCV, the analyses revealed presence of vesicles involved in ER to Golgi transport, Golgi membranes and various types of endocytic compartments. For instance, several components of COPI and COPII-coated vesicles, including CopA, CopG1 (Cop I) and Sec23A and Sar1A (COPII) were found to be part of the SMM proteome. In addition to components associated with the anterograde and retrograde transport system, several proteins involved in ER dynamics such as the small GTPase Rab10 and Rab2a, ER chaperones (protein disulfide-isomerase PDIA1, PDIA3, PDIA6, endoplasmin, hypoxia up-regulated protein 1), SNARE proteins (VAMP-associated protein A and B), vesicle recognition particles (subunit Srp72 and receptor SrpR), B-cell receptor associated protein 31, as well as diverse ER membrane proteins (e.g., transitional ER ATPase, calnexin, cytochrome B5, estradiol 17-beta dehydrogenase, dolichyl-diphospho-oligosaccharide-glycosyltransferase, transmembrane protein 43) were identified, hinting at a direct interaction of SMM with the ER system. Thus, the proteome inventory of SMM (Vorwerk et al., 2015) and observations from other studies indicate that intracellular *Sen* induce the fusion of various types of host cell compartments to the SCV and SIT (Drecktrah et al., 2008; Krieger et al., 2014). Furthermore, components of the actin cytoskeleton were identified as well as various proteins acting as linkers between F-actin and membrane compartments. In summary, these results indicate that SMM proteomics is a powerful approach to investigate molecular mechanisms of *Salmonella*-host interactions and to develop new intervention strategies.

By taking control over the host cell's vesicle fusion machinery, intracellular *Sen* create an extensive interconnected system of vesicles. On the one hand, this network is continuously interacting with incoming endosomal cargo, providing nutritional supply for the intracellular pathogen within SCVs connected to this network. On the other hand, for those SCV connected to the network, the large lumen results in rapid dilution of antimicrobial activities delivered during endosomal maturation to the SCV. This way, the tubular membrane network fulfills dual requirements for nutrition and protection against host defense mechanisms.

## Comparative proteomics of pathogen compartments

Intracellular development and replication of *Legionella*, *Mycobacterium*, *Chlamydia*, *Simkania*, and *Salmonella* spp. follow different routes, and the bacteria form unique pathogen-containing vacuoles (PCVs) (Figure 2). The varying localizations, characteristics and knowledge of the distinct PCVs required different approaches for purification of pathogen-modified vacuoles and membranes (Figure 3). For LCVs and SMMs vacuole-specific pathogen markers were exploited, i.e., the bacterial effector proteins SidC or SseF, which allowed compartment enrichment by immuno-magnetic separation based on distinct PCV components. The isolation of TDM bead phagosomes also included a magnetic separation step; however, in this case magnetic dynabeads covalently coated with BSA and TDM rather than the whole pathogen or a labeled PCV were enriched. For LCV and TDM bead phagosome purification density gradient centrifugation was used as a second purification step. Finally, for the purification of SMM differential centrifugation was used as an additional purification step, and the enrichment of CCVs or SnCVs solely relied on this separation principle.

Despite the distinct intracellular life-styles and the different technical approaches used, we were able to identify common host factors pointing to common strategies and interactions between cellular organelles and PCVs. Importantly, while the purification approaches were different for the pathogens, the preparations were mainly analyzed on the same proteomics platform. The technical consistency at the level of proteomics analysis is a major asset for the studies summarized here. The standardized analytical pipeline allowed drawing conclusions about PCV formation of the pathogens studied and likely also others.

**Figure 4:**
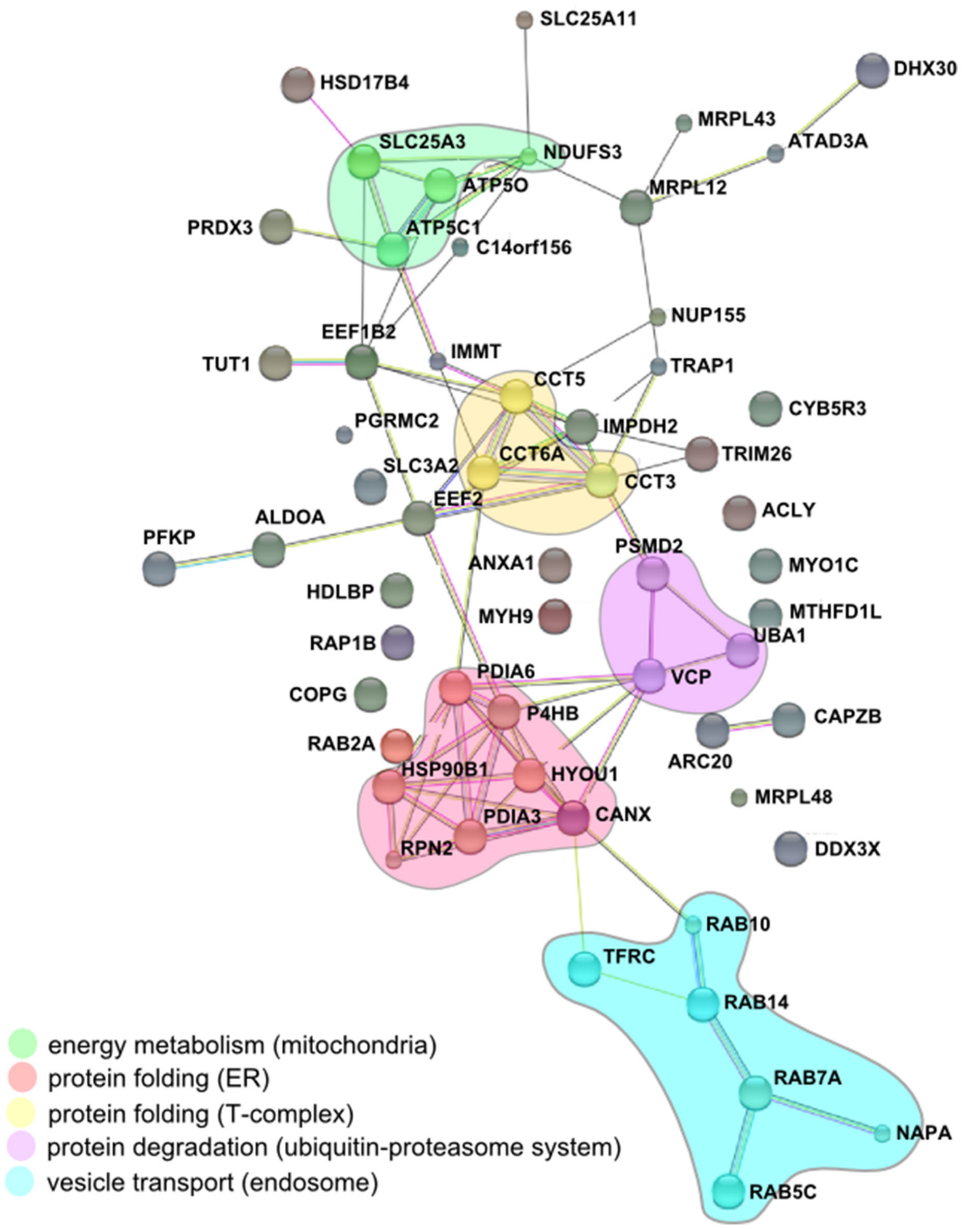
**Conserved components of LCVs, CCVs, SnCVs, and SMMs visualized using the STRING (Search Tool for Retrieval of Interacting Genes/Proteins) algorithm**. Marked (circles) are clusters comprised of interacting components from energy metabolism (mitochondria), protein folding (proteasome), protein folding (ER) and vesicle transport (endosomes). Lines represent protein-protein interactions with different layers of evidence: purple: experimental; blue: co-occurrence; red: fusion; black: co-expression; light blue: database; yellow: text-mining; green: neighborhood.

The comparison of the proteomic data obtained from enriched LCVs, SMM, CCVs, SnCVs and *Mycobacterium* TDM-bead phagosomes yielded a list of 34 common host factors (Table 1). The presence of host protein components of endosomal vesicle transport, protein folding and turnover (ER, T-complex, and ubiquitin-proteasome) as well as mitochondrial energy metabolism could be confirmed (Figure 4). Interestingly, more common host proteins were associated with LCVs and SnCVs (134 proteins, Table S1), SMM and SnCVs (183 proteins, Table S2) (Vorwerk et al., 2015), or LCVs, SMM and SnCVs (85 proteins, Table S3), compared with LCVs, SMM and CCVs (57 proteins, Table S4). Thus, it seems that in the case of *Legionella*, *Simkania*, and *Salmonella* the formation of the PCV is more connected to intracellular vesicle transport processes, including endosomal, clathrin-dependent, ER-derived, and Golgi-mediated pathways (Figure 4). Of note, we identified many similarities between LCVs and SnCVs, presumably because of their intimate interactions with the ER (Table S1). Since the SnCV is much closer connected to the ER than the LCV, unique host factors are likely to play additional roles.

**Table 1:** **MS-identified proteins observed in *Legionella*-containing vacuoles (LCV), *Salmonella*-modified membranes (SMM), *Simkania*-containing vacuoles-ER-membranes (SnCV-ER), *Chlamydia*-containing vacuoles (CCV) and *Mycobacterium*-phagosomes (TDM)**.

**#**	**Accession UniProtAC human**	**Protein description**	**Accession UniProtID human^1^**	**Accession UniProtID mouse^2^**	**Accession UniProtID dicti^3^**	**Detection HeLa SnCV**	**Detection THP1 SnCV**	**Detection HeLa CCV**	**Detection RAW 264.7 TDM**
1	ACLY	ATP-citrate synthase	P53396		Q54YA0	+	+	+	
2	ALDOA	Fructose-bisphosphate aldolase A	P04075		Q86A67	+	+	+	
3	ARPC4	Actin-related protein 2/3 complex subunit 4	P59998	P59999	O96625		+	+	+
4	C1TM	Monofunctional C1-tetrahydrofolate synthase, mitochondrial	Q6UB35	Q3V3R1		+		+	+
5	CAPZB	F-actin-capping protein subunit beta	P47756	P47757		+	+	+	
6	DHB4	Peroxisomal multifunctional enzyme type 2	P51659	P51660	Q9NKW1	+	+	+	+
7	DHX30	Putative ATP-dependent RNA helicase DHX30	Q7L2E3	Q99PU8		+		+	
8	EF1B	Elongation factor 1-beta	P24534	O70251	Q9GRF8	+	+	+	
9	EF1G	Elongation factor 1-gamma	P26641	Q9D8N0		+	+	+	
10	EF2	Elongation factor 2	P13639	P58252	P15112	+	+	+	+
11	IMDH2	Inosine-5′-monophosphate dehydrogenase 2	P12268	P24547		+	+	+	
12	MYH9	Myosin-9	P35579	Q8VDD5		+	+	+	+
13	PSMD2	26S proteasome non-ATPase regulatory subunit 2	Q13200	Q8VDM4		+	+	+	+
14	RM12	39S ribosomal protein L12, mitochondria	P52815	Q9DB15	Q86KA1	+	+	+	
15	RM43	39S ribosomal protein L43, mitochondrial	Q8N983	Q5RL20		+		+	
16	RM48	39S ribosomal protein L48, mitochondrial	Q96GC5	Q8JZS9		+		+	
17	SLIRP	SRA stem-loop-interacting RNA-binding protein, mitochondrial	Q9GZT3		Q86I7L	+		+	
18	TCPE	T-complex protein 1 subunit epsilon	P48643	P80316		+	+	+	
19	TCPG	T-complex protein 1 subunit gamma	P49368	P80318		+	+	+	+
20	TCPZ	T-complex protein 1 subunit zeta	P40227	P80317		+	+	+	
21	TERA	Transitional endoplasmic reticulum ATPase	P55072	Q01853	P90532	+	+	+	
22	TIF1B	Transcription intermediary factor 1-beta	Q13263	Q62318		+	+	+	+
23	DDX3X	ATP-dependent RNA helicase DDX3X	O00571	Q62167	Q54QS3	+	+	+	+
24	MYO1C	Myosin-Ic	O00159	Q9WTI7		+		+	
25	ANXA1	Annexin A1	P04083	P10107		+	+	+	+
26	ATD3A	ATPase family AAA domain-containing protein 3A	Q9NVI7	Q925I1		+		+	
27	ATPG	ATP synthase subunit gamma, mitochondrial	P36542	Q91VR2	Q54DF1	+	+	+	+
28	ATPO	ATP synthase subunit O, mitochondrial	P48047	Q9DB20	Q54RA8	+	+	+	
29	CALX	Calnexin	P27824	P35564	Q55BA8	+	+	+	+
30	COPG1	Coatomer subunit gamma -1	Q9Y678	Q9QZE5		+		+	+
31	ENPL	Endoplasmin	P14625	P08113		+	+	+	+
32	HYOU1	Hypoxia up-regulated protein 1	Q9Y4L1	Q9JKR6	Q556U6				
33	IMMT	Mitochondrial inner membrane protein	Q16891	Q8CAQ8			+	+	+
34	M2OM	Mitochondrial 2-oxoglutarate/malate carrier protein	Q02978	Q9CR62			+	+	
35	MPCP	Phosphate carrier protein, mitochondrial	Q00325	Q8VEM8	Q54BF6	+		+	+
36	NB5R3	NADH-cytochrome b5 reductase 3	P00387	Q9DCN2		+	+	+	+
37	NDUS3	NADH dehydrogenase [ubiquinone] iron-sulfur protein 3	O75489	Q9DCT2	P22237		+	+	+
38	NU155	Nuclear pore complex protein Nup155	O75694	Q99P88		+		+	+
39	PDIA1	Protein disulfide-isomerase	P07237	P09103		+	+	+	+
40	PDIA3	Protein disulfide-isomerase A3	P30101	P27773	Q54EN4	+	+	+	+
41	PDIA6	Protein disulfide-isomerase A6	Q15084		Q869Z0	+	+	+	+
42	PRDX3	Thioredoxin-dependent peroxide reductase, mitochondrial	P30048	P20108		+	+	+	+
43	RAB10	Ras-related protein Rab-10	P61026	P61027		+	+	+	+
44	RAB14	Ras-related protein Rab-14	P61106	Q91V41	P36410	+	+	+	+
45	RAB2A	Ras-related protein Rab-2A	P61019	P53994	P36409	+	+	+	+
46	RAB5C	Ras-related protein Rab-5C	P51148	P35278		+	+	+	+
47	RAB7A	Ras-related protein Rab-7a	P51149	P51150	P36411	+	+	+	+
48	RAP1B	Ras-related protein Rap-1b	P61224	Q99JI6	P18613	+	+	+	+
49	RPN2	Dolichyl-diphosphooligosaccharide-protein glycosyltransferase subunit 2	P04844	Q9DBG6		+	+	+	
50	SNAA	Alpha-soluble NSF attachment protein	P54920	Q9DB05		+	+	+	+
51	TFR1	Transferrin receptor protein 1	P02786	Q62351		+	+	+	+
52	VIGLN	Vigilin	Q00341	Q8VDJ3		+	+	+	
53	4F2	4F2 cell-surface antigen heavy chain	P08195	P10852		+	+	+	+
54	PGRC2	Membrane-associated progesterone receptor component 2	O15173	Q80UU9		+	+	+	
55	K6PP	6-phosphofructokinase type C	Q01813	Q9WUA3		+	+	+	
56	UBA1	Ubiquitin-like modifier-activating enzyme 1	P22314	Q02053		+	+	+	+

*1,SMM proteins isolated from HeLa (data set: (1)); 2,LCV proteins isolated from RAW 264.7 macrophages (data set: (2); 3,LCV proteins isolated from Dictyostelium strains (combined data sets: (2, 3))*.

1. *Vorwerk, S., Krieger, V J., Hensel, M., and Hansmeier N. (2014) Proteomes of host cells membranes modified by intracellular activities of Salmonella enterica. Mol. Cell. Proteomics 14(1), 81-92*.

2. *Hoffmann, C., Finsel, I., Otto, A., Pfaffinger, G., Rothmeier, E., Hecker, M., et al. (2013) Functional analysis of novel Rab GTPases identified in the proteome of purified Legionella-containing vacuoles from macrophages. Cell. Microbiol 16, 1034-52*.

3. *Shevchuk, O., Batzilla, C., Hagele, S., Kusch, H., Engelmann, S., Hecker, M., et al. (2009) Proteomic analysis of Legionella-containing phagosomes isolated from Dictyostelium. Int. J. Med. Microbiol 299, 489-508*.

The impact of ER-dependent protein folding on all PCVs may be reflected in the close association between ER and the PCV membranes (Gagnon et al., 2002; Tailleux et al., 2003; Isberg et al., 2009; Derre et al., 2011; Mehlitz et al., 2014). Furthermore, the association of some PCVs (LCVs, CCVs, SnCVs) with mitochondria indicated by microscopy and the identification of several mitochondrial proteins is peculiar and may indicate the common requirement of these intracellular pathogens to acquire metabolites from these organelles. Association with mitochondria was already described for *Lp*, *Ctr*, and *Sn*, which appear to recruit these compartments to the PCV (Horwitz, 1983; Matsumoto et al., 1991; Abu Kwaik, 1996; Tilney et al., 2001; Mehlitz et al., 2014). In summary, while due to their formation processes different PCVs comprise a unique set of components, some host factors as well as the communication with cell organelles is similar. The composition of a variety of purified PCVs described in this work represents a comprehensive inventory of eukaryotic and bacterial factors putatively implicated in PCV formation. The challenge lying ahead is the functional characterization of the host and pathogen components and their interactions in space and time.

Our studies led to the identification of host cell factors manipulated only by certain pathogens, as well as host factors that are commonly involved in the formation of PCV by clinically important intracellular bacteria. The latter group may comprise interesting new target structures for new strategies to interfere with intracellular proliferation. The knowledge of common mechanisms used for establishing an intracellular niche could provide new clues how to tackle the intracellular replication of important bacterial pathogens.

### Conflict of interest statement

The authors declare that the research was conducted in the absence of any commercial or financial relationships that could be construed as a potential conflict of interest.
