# Supplementary material for: Purification and proteomics of pathogen-modified vacuoles and membranes
Source: Front Cell Infect Microbiol. 2015 Jun 2;5:48. doi: 10.3389/fcimb.2015.00048 (PMC4451638; doi:10.3389/fcimb.2015.00048)
Supplement: Supplementary file 1 [file Table1.DOCX]

**Table S1.** MS-identified proteins observed in *Legionella* containing vacuoles (LCV) and *Simkania*-containing vacuoles-ER-membranes (SnCV-ER).

| **#** | **Protein** | **Protein description** | **Accession UniProtID human** | **Detection HeLa SnCV** | **Detection THP1 SnCV** | **Gene** | **Gene product** | **Accesion NCBI Gene mouse** | **Accesion NCBI Gene dicti** | **Localization** | **Function** |
| --- | --- | --- | --- | --- | --- | --- | --- | --- | --- | --- | --- |
| 1 | ACTN4 | Alpha-actinin-4 | O43707 | + | + | *AbpA* | alpha-Actinin-4 | NP_068695 (2) |  | CP | cytosceleton /motor proteins |
| 2 | ACTB (+1) | Actin, cytoplasmic 1 / 2 | P60709/P63261 | + | + | *ActA* | Actin | NP_033739 (3) |  | CP | cytosceleton /motor proteins |
| 3 | ARPC2 | Actin-related protein 2/3 complex subunit 2 | O15144 | + | + | *ArpC* | Arp2/3 complex, subunit | NP_083987 (3) |  | CP | cytosceleton /motor proteins |
| 4 | ARPC1B | Actin-related protein 2/3 complex subunit 1B | O15143 |  | + | *ArpC* | Arp2/3 complex, subunit | NP_083987 (3) |  | CP | cytosceleton /motor proteins |
| 5 | COR1A | Coronin-1A | P31146 |  | + | *CorA* | Coronin |  | DDB0191115 | EE | cytosceleton /motor proteins |
| 6 | COR1B | Coronin-1B | Q9BR76 |  | + | *CorA* | Coronin |  | DDB0191116 | EE | cytosceleton /motor proteins |
| 7 | COR1C | Coronin-1C | Q9ULV4 | + | + | *CorA* | Coronin |  | DDB0191117 | EE | cytosceleton /motor proteins |
| 8 | CORO7 | Coronin-7 | P57737 |  | + | *CorA* | Coronin |  | DDB0191118 | EE | cytosceleton /motor proteins |
| 9 | DC1L2 | Cytoplasmic dynein 1 light intermediate chain 2 | O43237 | + |  | *Dync1/2* | Dynein 1 intermediate chain 2 | NP_034194 (1) |  | CP | cytosceleton /motor proteins |
| 10 | IMA2 | Importin subunit alpha-2 | P52292 | + |  | *Kpna2* | Importin a2, isoform 1 | NP_034785 (1) |  | NC | cytosceleton /motor proteins |
| 11 | KTN1 | Kinectin | Q86UP2 | + | + | *Ktn1* | Kinectin | NP_032503 (3) |  | ER | cytosceleton /motor proteins |
| 12 | LBR | Lamin-B receptor | Q14739 |  | + | *Lbr* | Lamin B receptor | NP_598576 (3) |  | PM, NC | cytosceleton /motor proteins |
| 13 | LMNB1 | Lamin-B1 | P20700 |  | + | *Lmnb1* | Lamin B1 | NP_034851 (3) |  | PM, NC | cytosceleton /motor proteins |
| 14 | MOES | Moesin | P26038 | + | + | *Msn* | Moesin | NP_034963 (3) |  | CP, PM | cytosceleton /motor proteins |
| 15 | MYH9 | Myosin-9 | P35579 | + | + | *Myh9* | Myosin 9 isoform 1 | NP_071855 (3) |  |  | cytosceleton /motor proteins |
| 16 | NUMA1 | Nuclear mitotic apparatus protein 1 | Q14980 | + | + | *Numa1* | Nuclear mitotic apparatus protein 1 | NP_598708 (1) |  | CP, NC | cytosceleton /motor proteins |
| 17 | PROF1 | Profilin-1 | P07737 | + | + | *ProA* | Profilin | NP_035202 (2) | DDB0191178 (1) | CP | cytosceleton /motor proteins |
| 18 | RADI | Radixin | P35241 | + | + | *Rdx* | Radixin | NP_033067 (1) |  | CP, PM | cytosceleton /motor proteins |
| 19 | SEPT2 | Septin-2 | Q15019 | + | + | *Sept2a* | Septin 2a GTPase | NP_035021 (3) |  | CP | cytosceleton /motor proteins |
| 20 | STMN1 | Stathmin | P16949 |  | + | *Stmn1* | Stathmin | NP_062615 (2) |  | CP | cytosceleton /motor proteins |
| 21 | TBA1B (+1) | Tubulin alpha-1B chain | P68363 | + | + | *Tuba1b* | alpha-Tubulin, isoform 1b | NP_035784 (3) |  | CP | cytosceleton /motor proteins |
| 22 | ALCAM | CD166 antigen | Q13740 |  | + | *Alcam* | Activated leukocyte cell adhesion molecule; CD166 | NP_061201 (3) |  | PM | immune response |
| 23 | ERAP1 | Endoplasmic reticulum aminopeptidase 1 | Q9NZ08 |  | + | *Erap1* | ER aminopeptidase 1 | NP_109636 (3) |  | ER | immune response |
| 24 | B2M | Beta-2-microglobulin | P61769 |  | + | *B2m* | Beta-2-microglobulin | NP_033865 (2) |  | PM | immune response |
| 25 | BCAP31 | B-cell receptor-associated protein 31 | P51572 |  | + | *Bcap31* | B-cell receptor-assoc. prot. 31 | NP_036190 (3) |  | PM | immune response |
| 26 | BSG | Basigin | P35613 |  | + | *Bsg* | Basigin isoform 2; CD147 | NP_001070652 (3) |  | PM | immune response |
| 27 | C1QBP | Complement component 1 Q subcomponent-binding protein, mitochondrial | Q07021 |  | + | *C1qbp* | Complement component 1 Q subcomponent-binding protein | NP_031599 (3) |  | PM | immune response |
| 28 | CD14 | Monocyte differentiation antigen CD14 | P08571 |  | + | *CD14* | Monocyte differentiationantigen CD14 | NP_033971 (3) |  | PM | immune response |
| 29 | CD44 | CD44 antigen | P16070 | + | + | *CD44* | CD44 antigen isoform d | NP_001171256 (2) |  | PM | immune response |
| 30 | CD63 | CD63 antigen | P08962 |  | + | *CD63* | CD63 antigen | NP_001036045 (1) |  | PM | immune response |
| 31 | CD74 | HLA class II histocompatibility antigen gamma chain | P04233 |  | + | *CD74* | H-2 MHC class II, y chain | NP_034675 (3) |  | PM | immune response |
| 32 | FUBP2 | Far upstream element-binding protein 2 | Q92945 | + | + | *Khrsp* | Far upstream element-binding protein 2 | NP_034743 (1) |  | CP, NC | immune response |
| 33 | HCLS1 | Hematopoietic lineage cell-specific protein | P14317 |  | + | *Hcls1* | Hematopoietic lineage cell-specific protein | NP_032251 (1) |  | PM, CP | immune response |
| 34 | HM13 | Minor histocompatibility antigen H13 | Q8TCT9 |  | + | *Hm13* | Minor histocompability antigen H13 isoform 2 | NP_034506 (3) |  | PM | immune response |
| 35 | TAP1 | Antigen peptide transporter 1 | Q03518 |  | + | *Tap1* | Antigen peptide transporter-1 | NP_038711 (3) |  | ER | immune response |
| 36 | ALOX5AP | Arachidonate 5-lipoxygenase-activating protein | P20292 |  | + | *Alox5ap* | Arachidonate 5-lipooxygenase activating protein | NP_033793 (1) |  | ER, GA | lipid metabolism |
| 37 | ASAH1 | Acid ceramidase | Q13510 |  | + | *Asah1* | Acid ceramidase | NP_062708 (3) |  | ER, GA | lipid metabolism |
| 38 | FDFT1 | Squalene synthase | P37268 |  | + | *FdfT* | Squalene synthase |  | DDB0231376 (2) | ER | lipid metabolism |
| 39 | GBA | Glucosylceramidase | P04062 |  | + | *Gba* | Glucosylceramidase isoform 2 | NP_001070879 (3) |  | LS | lipid metabolism |
| 40 | NCEH1 | Neutral cholesterol ester hydrolase 1 | Q6PIU2 | + | + | *Nceh1* | Neutral cholesterol ester hydrolase 1 | NP_848887 (3) |  | ER | lipid metabolism |
| 41 | OSBPL8 | Oxysterol-binding protein-related protein 8 | Q9BZF1 |  | + | *Osbpl8* | Oxysterol-binding protein-like protein 8, isoform a (OsbH) | NP_780698 (1) |  | ER, GA | lipid metabolism |
| 42 | PGRC1 | Membrane-associated progesterone receptor component 1 | O00264 | + |  | *Pgrmc1* | Membrane assoc. progesterone receptor component 1 | NP_058063 (3) |  | ER | lipid metabolism |
| 43 | PPT1 | Palmitoyl-protein thioesterase 1 | P50897 |  | + | *Ppt1* | Palmitoyl protein thioesterase 1 | NP_032943 (3) |  | LS | lipid metabolism |
| 44 | PGES2 | Prostaglandin E synthase 2 | Q9H7Z7 | + |  | *Ptges2* | Prostaglandin E synthase 2 | NP_598544 (3) |  | CP, GA, NC | lipid metabolism |
| 45 | NLTP | Non-specific lipid-transfer protein | P22307 | + | + | *Scp2* | Non-specific lipid-transfer protein | NP_035457 (3) |  | CP, MC | lipid metabolism |
| 46 | ZMPSTE24 | CAAX prenyl protease 1 homolog | O75844 |  | + | *Zmpste24* | CAAX prenyl protease 1 | NP_766288 (1) |  | ER, GA | lipid metabolism |
| 47 | ARF1 | ADP-ribosylation factor 1 | P84077 |  | + | *Arf1* | Arf1 GTPase (ArfA) | NP_001123880 (2) | DDB0191101 (2) | GA, LCV | membrane dynamics |
| 48 | CLH1 | Clathrin heavy chain 1 | Q00610 | + | + | *Cltc* | Clathrin heavy chain (ChcA) | NP_001003908 | DDB0185029 (3) | PM, EE | membrane dynamics |
| 49 | COPA | Coatomer subunit alpha | P53621 | + | + | *CopA* | Coatomer subunit alpha | NP_034068 (1) |  | CP, GA | membrane dynamics |
| 50 | EHD1 | EH domain-containing protein 1 | Q9H4M9 |  | + | *Ehd1* | EH domain-containing protein 1 | NP_034249 (3) |  | RE | membrane dynamics |
| 51 | MYOF | Myoferlin | Q9NZM1 | + |  | *Myof* | Myoferlin | NP_001093104 (2) |  | PM, NC | membrane dynamics |
| 52 | NAPA | Alpha-soluble NSF attachment protein | P54920 |  | + | *Napa* | alpha-soluble NSF attachment protein (SnpA) | NP_080174 (2) |  | CP | membrane dynamics |
| 53 | RAB1A | Ras-related protein Rab-1A | P62820 | + | + | *Rab1A* | Ras GTPase-related protein (Rab) 1A | BAF02845 (1) | DDB0191476 (3) | ER, GA, LCV | membrane dynamics |
| 54 | RAB1B | Ras-related protein Rab-1B | Q9H0U4 |  | + | *Rab1B* | Ras GTPase-related protein (Rab) 1B | BAF02846 (3) |  | ER,GA | membrane dynamics |
| 55 | RAB2A | Ras-related protein Rab-2A | P61019 | + | + | *Rab2A* | Ras GTPase-related protein (Rab) 2A | BAF02847 (1) | DDB0216191 (3) | ER, GA | membrane dynamics |
| 56 | RAB2B | Ras-related protein Rab-2B | Q8WUD1 |  | + | *Rab2B* | Ras GTPase-related protein (Rab) 2B |  | DDB0229402 (1) |  | membrane dynamics |
| 57 | RAB5A | Ras-related protein Rab-5A | P20339 | + | + | *Rab5A* | Ras GTPase-related protein (Rab) 5A | NP_080163 (1) | DDB0229401 (1) | EE | membrane dynamics |
| 58 | RAB5C | Ras-related protein Rab-5C | P51148 | + | + | *Rab5C* | Ras GTPase-related protein (Rab) 5C | BAF02857 (1) |  | EE | membrane dynamics |
| 59 | RAB6A | Ras-related protein Rab-6A | P20340 | + | + | *Rab6A* | Ras GTPase-related protein (Rab) 6A | BAF02858 (1) |  | GA, ERGIC | membrane dynamics |
| 60 | RAB7A | Ras-related protein Rab-7a | P51149 | + | + | *Rab7A* | Ras GTPase-related protein (Rab) 7A | NP_033031 (3) | DDB0191507 (2) | LE, LS, LCV | membrane dynamics |
| 61 | RAB8A | Ras-related protein Rab-8A | P61006 | + | + | *Rab8A* | Ras GTPase-related protein (Rab) 8A | BAF02862 (1) | DDB0214885 (3) | PM, RE, GA, LCV | membrane dynamics |
| 62 | RAB10 | Ras-related protein Rab-10 | P61026 | + | + | *Rab10* | Ras GTPase-related protein (Rab) 10 | CAJ18601 (1) |  | GA | membrane dynamics |
| 63 | RAB11A | Ras-related protein Rab-11A | P62491 |  | + | *Rab11A* | Ras GTPase-related protein (Rab) 11A | BAf02867 (3) | DDB0191190 (3) | RE, GA | membrane dynamics |
| 64 | RAB14 | Ras-related protein Rab-14 | P61106 | + | + | *Rab14* | Ras GTPase-related protein (Rab) 14 | BAF02871 (1) | DDB0214821 (3) | EE, LCV, MCV | membrane dynamics |
| 65 | RAB18 | Ras-related protein Rab-18 | Q9NP72 | + | + | *Rab18* | Ras GTPase-related protein (Rab) 18 | AAC37632 (1) | DDB0229409 (1) | ER | membrane dynamics |
| 66 | RAB21 | Ras-related protein Rab-21 | Q9UL25 | + | + | *Rab21* | Ras GTPase-related protein (Rab) 21 | BAF02877 (1) |  | EE | membrane dynamics |
| 67 | RAB31 | Ras-related protein Rab-31 | Q13636 | + | + | *Rab31* | Ras GTPase-related protein (Rab) 31 | NP_598446 (2) |  | EE, GA | membrane dynamics |
| 68 | RAB32 | Ras-related protein Rab-32 | Q13637 | + |  | *Rab32A* | Ras GTPase-related protein (Rab) 32A |  | DDB0201639 (1) | MC | membrane dynamics |
| 69 | RTN4 | Reticulon-4 | Q9NQC3 | + | + | *Rtn4* | Reticulon-4, isoform A (Nogo) | NP_918943 (2) |  | PM, ER | membrane dynamics |
| 70 | SAR1A | GTP-binding protein SAR1a | Q9NR31 | + |  | *SarA* | SarA GTPase |  | DDB0229965 (2) | ER, ERGIC, LCV | membrane dynamics |
| 71 | SCAMP3 | Secretory carrier-associated membrane protein 3 | O14828 |  | + | *Scamp3* | Secretory carrier-associated membrane protein 3 | O35609 (3) |  | LE, LS | membrane dynamics |
| 72 | SDCB1 | Syntenin-1 | O00560 | + | + | *Sdcbp* | Syntenin 1, isoform 2 (Mda9) | NP_058087 (2) |  | PM, EE | membrane dynamics |
| 73 | SC22B | Vesicle-trafficking protein SEC22b | O75396 | + | + | *Sec22B* | Sec22b v-SNARE | NP_035472 (3) |  | ER, LCV | membrane dynamics |
| 74 | SNP23 | Synaptosomal-associated protein 23 | O00161 | + | + | *Snap23* | Synaptosomal-associated protein 23 isoform a | NP_001171263 (1) |  | PM | membrane dynamics |
| 75 | SNX2 | Sorting nexin-2 | O60749 |  | + | *Snx2* | Sorting nexin 2 | NP_080662 (1) |  | CP, EE | membrane dynamics |
| 76 | SNX5 | Sorting nexin-5 | Q9Y5X3 |  | + | *Snx5* | Sorting nexin 5 | NP_035566 (1) |  | CP, EE | membrane dynamics |
| 77 | STX4 | Syntaxin-4 | Q12846 | + | + | *Stx4* | Syntaxin 4 t-SNARE | NP_033320 (2) |  | PM | membrane dynamics |
| 78 | STX7 | Syntaxin-7 | O15400 |  | + | *Stx7* | Syntaxin 7 t-SNARE | NP_058077 (3) |  | PM | membrane dynamics |
| 79 | SYNGR1 | Synaptogyrin-1 | O43759 |  | + | *Syngr1* | Synaptogyrin 1 | NP_997591 (2) |  | PM | membrane dynamics |
| 80 | SYJ2B | Synaptojanin-2-binding protein | P57105 | + |  | *Synj2bp* | Synaptojanin-2-binding protein | NP_079568 (2) |  | CP, PM, MC | membrane dynamics |
| 81 | TFR1 | Transferrin receptor protein 1 | P02786 | + | + | *Tfrc* | Transferrin receptor protein 1 | NP_035768 (3) |  | PM, EE | membrane dynamics |
| 82 | VAMP3 | Vesicle-associated membrane protein 3 | Q15836 | + | + | *Vamp3* | Vesicle-associated membrane protein 3 v-SNARE | NP_033524 (2) |  | LE | membrane dynamics |
| 83 | VAMP4 | Vesicle-associated membrane protein 4 | O75379 |  | + | *Vamp4* | Vesicle-associated membrane protein 4 v-SNARE | NP_058076 (2) |  | PM, EE | membrane dynamics |
| 84 | VAMP7 | Vesicle-associated membrane protein 7 | P51809 | + | + | *Vamp7* | Vesicle-associated membrane protein 7 v-SNARE | NP_035645 (1) | DDB0231542 (1) | PM, EE | membrane dynamics |
| 85 | VAMP8 | Vesicle-associated membrane protein 8 | Q9BV40 | + | + | *Vamp8* | Vesicle-associated membrane protein 8 v-SNARE | NP_058074 (2) |  | LE | membrane dynamics |
| 86 | VAPA | Vesicle-associated membrane protein-associated protein A | Q9P0L0 |  | + | *VapA* | Vamp-associated protein A | NP_038961 (2) |  | ER | membrane dynamics |
| 87 | LMAN2 | Vesicular integral-membrane protein VIP36 | Q12907 | + | + | *Vip36* | Vesicular integral membrane protein (Lman2 lectin) | NP_080104 (3) |  | ER | membrane dynamics |
| 88 | ANXA1 | Annexin A1 | P04083 | + | + | *Anxa1* | Annexin A1 | NP_034860 (2) |  | CP, NC | miscellaneous |
| 89 | CAT | Catalase | P04040 |  | + | *CatA* | Catalase | NP_033934 (3) | DDB0185123 (3) | MC | miscellaneous |
| 90 | CALX | Calnexin | P27824 | + | + | *CnxA* | Calnexin | AAH40244 (1) | DDB0215348 (3) | ER, LCV | miscellaneous |
| 91 | CALR | Calreticulin | P27797 | + | + | *CrtA* | Calreticulin | NP_031617 (3) | DDB0191384 (3) | ER, LCV | miscellaneous |
| 92 | CTSZ | Cathepsin Z | Q9UBR2 |  | + | *CtsZ* | Cathepsin Z | NP_071720 (3) | DDB0233836 (1) | LE, LS | miscellaneous |
| 93 | GSLG1 | Golgi apparatus protein 1 | Q92896 | + |  | *Glg1* | Golgi apparatus protein 1 | NP_033175 |  | PM, GA | miscellaneous |
| 94 | LAMP1 | Lysosome-associated membrane glycoprotein 1 | P11279 | + | + | *Lamp1* | Lysosome-associated membrane glycoprotein 1 | NP_034814 (3) |  | LE, LS | miscellaneous |
| 95 | LMAN1 | Protein ERGIC-53 | P49257 | + | + | *Lman1* | ERGIC53 | NP_081676 (3) |  | ERGIC | miscellaneous |
| 96 | MAN2B1 | Lysosomal alpha-mannosidase | O00754 |  | + | *Man2b1* | Lysosomal alpha-mannosidase | NP_034894 (1) |  | LS | miscellaneous |
| 97 | PDIA1 | Protein disulfide-isomerase | P07237 | + | + | *Pdi1* | Protein disulfide isomerase | NP_035162 (3) | DDB0185040 (3) | ER | miscellaneous |
| 98 | PRDX1 | Peroxiredoxin-1 | Q06830 | + | + | *Prdx1* | Peroxiredoxin (AhpC) | NP_035164 | DDB0238006 (3) | MC | miscellaneous |
| 99 | SC11A | Signal peptidase complex catalytic subunit SEC11A | P67812 | + | + | *Sec11* | Signal sequence peptidase | NP_064335 (2) | DDB0237791 (2) | ER | miscellaneous |
| 100 | S61A1 | Protein transport protein Sec61 subunit alpha isoform 1 | P61619 | + |  | *Sec61a* | Sec 61 channel | NP_058602 (3) | DDB0235194 (3) | ER | miscellaneous |
| 101 | AIFM1 | Apoptosis-inducing factor 1, mitochondrial | O95831 |  | + | *Aif* | Apoptosis-inducing factor (PCD protein 8, isoform 1) | NP_036149 (3) | DDB0191137 (3) | MC | signaling |
| 102 | ARHG2 | Rho guanine nucleotide exchange factor 2 | Q92974 | + | + | *Arhgef2* | Rho GEF H1 | NP_032513 (1) |  | PM | signaling |
| 103 | ARHGDIA | Rho GDP-dissociation inhibitor 1 | P52565 |  | + | *Arhgdia* | Rho GDI 1 | NP_598557 (1) |  | PM | signaling |
| 104 | BAX | Apoptosis regulator BAX | Q07812 |  | + | *Bax* | Apoptosis regulator Bax | NP_031553 (3) |  | CP, MC, ER | signaling |
| 105 | BRI3BP | BRI3-binding protein | Q8WY22 |  |  | *Bri3bp* | Bri3-binding protein | NP_084028 (3) |  | ER | signaling |
| 106 | CDK1 | Cyclin-dependent kinase 1 | P06493 | + | + | *Cdk1* | Cyclin-dependent kinase 1 | NP_031685 (1) | DDB0185028 (1) | CP, MC, NC | signaling |
| 107 | CDK5RAP3 | CDK5 regulatory subunit-associated protein 3 | Q96JB5 |  | + | *Cdk5rap3* | CDK5 regulatory subunit-associated protein 3 | NP_084524 (3) |  | PM | signaling |
| 108 | IQGA1 | Ras GTPase-activating-like protein IQGAP1 | P46940 | + | + | *GapA* | IQGAP-related Ras GAP |  | DDB0233055 (1) | CP | signaling |
| 109 | GRAP | GRB2-related adapter protein | REVERSE_Q13588 |  |  | *Grb2* | Growth factor receptor-bound protein 2 | NP_032189 (1) |  | PM | signaling |
| 110 | ITGA4 | Integrin alpha-4 | P13612 |  | + | *Itga4* | Integrin alpha 4 | NP_034706 (3) |  | PM | signaling |
| 111 | LTOR1 | Ragulator complex protein LAMTOR1 | Q6IAA8 | + | + | *LAMTOR1* | RhoA activator C11 homolouge | NP_079881 (2) |  | PM | signaling |
| 112 | LYN | Tyrosine-protein kinase Lyn | P07948 |  | + | *Lyn* | Tyr prot. kinase Lyn isoform B | NP_034877 (3) |  | PM | signaling |
| 113 | MARCKSL1 | MARCKS-related protein | P49006 |  | + | *Marcksl1* | MARCKS-related protein 1 | NP_034937 (3) |  | PM | signaling |
| 114 | PDCD6 | Programmed cell death protein 6 | O75340 | + |  | *Pdcd6ip* | Programmed cell death 6-interacting protein, isoform 1 | NP_001158149 (2) |  | CP | signaling |
| 115 | PHB2 | Prohibitin-2 | Q99623 | + | + | *PhbA* | Prohibitin | NP_032857 (1) | DDB0232063 (3) | CP, MC, NC | signaling |
| 116 | PPP2R1A | Serine/threonine-protein phosphatase 2A 65 kDa regulatory subunit A alpha isoform | P30153 |  | + | *Ppp2r1a* | Ser/Thr protein phosphatase 2, regulatory subunit A-alpha | NP_058587 (1) |  | CP, NC | signaling |
| 117 | RBP2 | E3 SUMO-protein ligase RanBP2 | P49792 | + |  | *Ranbp2* | E3 SUMO-prot. Ligase RanBP2 | NP_035370 (1) |  | NC | signaling |
| 118 | RCC1 | Regulator of chromosome condensation | P18754 | + |  | *Rcc* | Regulator of chromosome condensation |  | DDB0235387 (2) | PM | signaling |
| 119 | PTPN6 | Tyrosine-protein phosphatase non-receptor type 6 | P29350 |  | + | *Ptpn6* | Tyr protein phosphatase non-receptor type 6 | NP_038573 (3) |  | CP, NC | signaling |
| 120 | PTPRC | Receptor-type tyrosine-protein phosphatase C | P08575 |  | + | *Ptprc* | Receptor-type Tyr protein phosphatase C isoform 2 | NP_035340 (3) |  | PM | signaling |
| 121 | SACM1L | Phosphatidylinositide phosphatase SAC1 | Q9NTJ5 |  | + | *Sac1* | PI phosphatase Sac1 | NP_109617 (3) | DDB0233948 (3) | ER | signaling |
| 122 | INPP5D | Phosphatidylinositol-3,4,5-trisphosphate 5-phosphatase 1 | Q92835 |  | + | *Ship1* | PtdIns(3,4,5)P3 5-phosphatase 1 | NP_034696 (1) |  | CP, PM | signaling |
| 123 | STAT1 | Signal transducer and activator of transcription 1-alpha/beta | P42224 |  | + | *Stat1* | Signal transducer and activator of transcription 1 | NP_033309 (1) |  | CP, NC | signaling |
| 124 | TRAP1 | Heat shock protein 75 kDa, mitochondrial | Q12931 |  | + | *Trap1* | TNFR-associated protein |  | DDB0185036 (3) | CP, PM | signaling |
| 125 | TYROBP | TYRO protein tyrosine kinase-binding protein OS=Homo sapiens GN=TYROBP PE=1 SV=1 | O43914 |  |  | *Tyrobp* | TYRO protein Tyr kinase-binding protein | NP_035792 (1) |  | PM | signaling |
| 126 | UBA1 | Ubiquitin-like modifier-activating enzyme 1 | P22314 | + |  | *Uba1* | Ubiquitin-like modifier-activating enzyme 1, isoform 2 | NP_001129557 (1) |  | CP, NC | signaling |
| 127 | 1433E | 14-3-3 protein epsilon | P62258 | + | + | *Ywhae* | 14-3-3 protein subunit epsilon | NP_033562 (3) |  | CP | signaling |
| 128 | ATP1A1 | Sodium/potassium-transporting ATPase subunit alpha-1 | P05023 |  | + | *Atp1a1* | Na^+^/K^+^ ATPase alpha-1 | NP_061201 (3) |  | PM | transport |
| 129 | ATP2A2 | Sarcoplasmic/endoplasmic reticulum calcium ATPase 2 | P16615 |  | + | *Atp2a2* | Ca^2+^ transport ATPase | NP_001103610 (3) |  | ER or PM | transport |
| 130 | ATP5A1 | ATP synthase subunit alpha, mitochondrial | P25705 |  | + | *Atp5a1* | ATP synthase | NP_031531 (3) |  | MC | transport |
| 131 | ATP6V1A | V-type proton ATPase catalytic subunit A | P38606 |  | + | *Atp6v1a* | V-type H^+^-ATPase (VatA) | NP_031534 (3) | DDB0201563 (3) | LE, LS | transport |
| 132 | AAAT | Neutral amino acid transporter B(0) | Q15758 | + | + | *Slc1a5* | Neutral amino acid transporter B | NP_033227 (3) |  | PM | transport |
| 133 | GTR1 | Solute carrier family 2, facilitated glucose transporter member 1 | P11166 | + | + | *Slc2a1* | Solute carrier family 2, faciliated glucose transporter 1 (Glut1) | NP_035530 (3) |  | PM | transport |
| 134 | VDAC1 | Voltage-dependent anion-selective channel protein 1 | P21796 | + | + | *Vdac1* | Voltage-dependent anion-selective channel protein 1 | NP_035824 (3) |  | PM, MC | transport |

**Table S2.** MS-identified proteins observed in *Salmonella*-modified membranes (SMM) and *Simkania*-containing vacuoles-ER-membranes (SnCV-ER).

| **#** | **Protein** | **Protein description** | **Accession UniProtID human** | **Detection HeLa SnCV** | **Detection THP1 SnCV** |
| --- | --- | --- | --- | --- | --- |
| 1 | 2AAA | Serine/threonine-protein phosphatase 2A 65 kDa | P30153 | + | + |
| 2 | 4F2 | 4F2 cell-surface antigen heavy chain | P08195 | + | + |
| 3 | ACLY | ATP-citrate synthase | P53396 | + | + |
| 4 | ACTN1 | Alpha-actinin-1 | P12814 | + | + |
| 5 | ADT3 | ADP/ATP translocase 3 | [P12236](http://www.uniprot.org/uniprot/P12236) | + |  |
| 6 | AHNK | Neutroblast differentiation-associated protein AHNAK | [Q09666](http://www.uniprot.org/uniprot/Q09666) | + | + |
| 7 | AIMP1 | Aminoacyl tRNA synthase complex-interacting multifunctional protein 1 | [Q12904](http://www.uniprot.org/uniprot/Q12904) | + | + |
| 8 | ALDOA | Fructose-bisphosphate aldolase A | P04075 | + | + |
| 9 | ANXA1 | Annexin A1 | P04083 | + | + |
| 10 | AP2A1 | AP-2 complex subunit alpha-1 | O95782 | + | + |
| 11 | AP2B1 | AP-2 complex subunit beta | [P63010](http://www.uniprot.org/uniprot/P63010) | + | + |
| 12 | ARF4 | ADP-ribosylation factor 4 | [P18085](http://www.uniprot.org/uniprot/P18085) | + | + |
| 13 | AROS | Active regulator of SIRT1 | [Q86WX3](http://www.uniprot.org/uniprot/Q86WX3) | + |  |
| 14 | ARPC4 | Actin-related protein 2/3 complex subunit 4 | P59998 | + | + |
| 15 | AT5F1 | ATP synthase subunit b, mitochondrial | P24539 | + | + |
| 16 | ATD3A | ATPase family AAA domain-containing protein 3A | Q9NVI7 | + |  |
| 17 | ATP5H | ATP synthase subunit d, mitochondrial | [O75947](http://www.uniprot.org/uniprot/O75947) | + | + |
| 18 | ATPG | ATP synthase subunit gamma, mitochondrial | P36542 | + | + |
| 19 | ATPO | ATP synthase subunit O, mitochondrial | P48047 | + | + |
| 20 | BAG2 | BAG family molecular chaperone regulator 2 | O95816 | + | + |
| 21 | BAP31 | B-cell receptor-associated protein 31 | P51572 | + | + |
| 22 | BOP1 | Ribosome biogenesis protein BOP1 | [Q14137](http://www.uniprot.org/uniprot/Q14137) | + | + |
| 23 | C1TM | Monofunctional C1-tetrahydrofolate synthase, mitochondrial | Q6UB35 | + |  |
| 24 | CALX | Calnexin | P27824 | + | + |
| 25 | CAPZB | F-actin-capping protein subunit beta | P47756 | + | + |
| 26 | CDC5L | Cell division cacle 5-like protein | [Q99459](http://www.uniprot.org/uniprot/Q99459) | + | + |
| 27 | CN166 | UPF0568 protein C14orf166 | [Q9Y224](http://www.uniprot.org/uniprot/Q9Y224) | + |  |
| 28 | COPA | Coatomer subunit alpha | P53621 | + | + |
| 29 | COPG1 | Coatomer subunit gamma -1 | Q9Y678 | + |  |
| 30 | COR1C | Coronin-1C | Q9ULV4 | + | + |
| 31 | CTNA1 | Catenin alpha | [P35221](http://www.uniprot.org/uniprot/P35221) | + |  |
| 32 | CUL4A | Cullin-4A | [Q13619](http://www.uniprot.org/uniprot/Q13619) | + | + |
| 33 | DDX1 | ATP-dependent RNA helicase DDX1 | [Q92499](http://www.uniprot.org/uniprot/Q92499) | + | + |
| 34 | DDX17 | Probable ATP-dependent RNA helicase DDX17 | [Q92841](http://www.uniprot.org/uniprot/Q92841) | + | + |
| 35 | DDX23 | Probable ATP-dependent RNA helicase DDX23 | [Q9BUQ8](http://www.uniprot.org/uniprot/Q9BUQ8) | + | + |
| 36 | DDX3X | ATP-dependent RNA helicase DDX3X | O00571 | + | + |
| 37 | DDX56 | Probable ATP-dependent RNA helicase DDX56 | [Q9NY93](http://www.uniprot.org/uniprot/Q9NY93) | + |  |
| 38 | DHB4 | Peroxisomal multifunctional enzyme type 2 | P51659 | + | + |
| 39 | DHX30 | Putative ATP-dependent RNA helicase DHX30 | Q7L2E3 | + |  |
| 40 | DSRAD | Double-stranded RNA-specific adenosine deaminase | [P55265](http://www.uniprot.org/uniprot/P55265) | + |  |
| 41 | DUT | Deoxyuridine 5'-triphosphate nucleotidohydrolase, mitochondrial | [P33316](http://www.uniprot.org/uniprot/P33316) | + | + |
| 42 | DX39A | ATP-dependent RNA helicase DDX39A | [O00148](http://www.uniprot.org/uniprot/O00148) | + |  |
| 43 | DYHC1 | Cytoplasmic dynein 1 heavy chain 1 | [Q14204](http://www.uniprot.org/uniprot/Q14204) | + |  |
| 44 | EF1B | Elongation factor 1-beta | P24534 | + | + |
| 45 | EF1G | Elongation factor 1-gamma | P26641 | + | + |
| 46 | EF2 | Elongation factor 2 | P13639 | + | + |
| 47 | EHD4 | EH domain-containing protein 4 | Q9H223 |  | + |
| 48 | ELAV1 | ELAV-like protein 1 | [Q15717](http://www.uniprot.org/uniprot/Q15717) | + |  |
| 49 | ENPL | Endoplasmin | P14625 | + | + |
| 50 | ESYT1 | Extended synaptotagmin-1 | [Q9BSJ8](http://www.uniprot.org/uniprot/Q9BSJ8) | + | + |
| 51 | EXOS2 | Exosome complex component RRP4 | [Q13868](http://www.uniprot.org/uniprot/Q13868) | + |  |
| 52 | EZRI | Ezrin | [P15311](http://www.uniprot.org/uniprot/P15311) | + | + |
| 53 | FAS | Fatty acid synthase | [P49327](http://www.uniprot.org/uniprot/P49327) | + | + |
| 54 | FLNA | Flamin-A | [P21333](http://www.uniprot.org/uniprot/P21333) | + | + |
| 55 | FXR1 | Fragile X mental retardation syndrome-related protein 1 | [P51114](http://www.uniprot.org/uniprot/P51114) | + | + |
| 56 | G3BP2 | Ras GTPase-activating protein-binding protein 2 | [Q9UN86](http://www.uniprot.org/uniprot/Q9UN86) | + | + |
| 57 | GALT2 | Polypeptide N-Acetylgalactosaminyltransferase 2 | [Q10471](http://www.uniprot.org/uniprot/Q10471) | + |  |
| 58 | GCN1L | Translational activator GCN1 | [Q92616](http://www.uniprot.org/uniprot/Q92616) | + | + |
| 59 | HDAC2 | Histone deacetylase 2 | [Q92769](http://www.uniprot.org/uniprot/Q92769) | + |  |
| 60 | HNRPD | Heterogeneous nuclear ribonucleoprotein D0 | [Q14103](http://www.uniprot.org/uniprot/Q14103) | + |  |
| 61 | HNRPL | Heterogeneous nuclear ribonucleoprotein L | [P14866](http://www.uniprot.org/uniprot/P14866) | + |  |
| 62 | HYOU1 | Hypoxia up-regulated protein 1 | Q9Y4L1 |  | + |
| 63 | IF1AY | Eukaryotic translation initiation factor 1A, Y-chromosomal | [O14602](http://www.uniprot.org/uniprot/O14602) | + |  |
| 64 | IMA4 | Importin subunit alpha-4 | [O00505](http://www.uniprot.org/uniprot/O00505) | + |  |
| 65 | IMDH2 | Inosine-5'-monophosphate dehydrogenase 2 | P12268 | + | + |
| 66 | IMMT | Mitochondrial inner membrane protein | Q16891 |  | + |
| 67 | IPO7 | Importin-7 | [O95373](http://www.uniprot.org/uniprot/O95373) | + |  |
| 68 | K0020 | Pumilio domain-containing protein KIAA0020 | [Q15397](http://www.uniprot.org/uniprot/Q15397) | + | + |
| 69 | K6PP | 6-phosphofructokinase type C | Q01813 | + |  |
| 70 | KHDR1 | KH domain-containing, RNA-binding, signal transduction-associated protein 1 | [Q07666](http://www.uniprot.org/uniprot/Q07666) | + |  |
| 71 | LAP2A | Lamina-associated polypeptide 2, isoform alpha | [P42166](http://www.uniprot.org/uniprot/P42166) | + |  |
| 72 | LDHA | L-lactate dehydrogenase A chain | [P00338](http://www.uniprot.org/uniprot/P00338) | + | + |
| 73 | LMNB2 | Lamin-B2 | Q03252 | + |  |
| 74 | LMO7 | LIM domain only protein 7 | [Q8WWI1](http://www.uniprot.org/uniprot/Q8WWI1) | + |  |
| 75 | M2OM | Mitochondrial 2-oxoglutarate/malate carrier protein | Q02978 |  | + |
| 76 | MGST | Microsomal glutathione S-transferase 1 | [P10620](http://www.uniprot.org/uniprot/P10620) | + |  |
| 77 | MPCP | Phosphate carrier protein, mitochondrial | Q00325 | + |  |
| 78 | MYH10 | Myosin-10 | P35580 | + |  |
| 79 | MYH9 | Myosin-9 | P35579 | + | + |
| 80 | MYO1C | Myosin-Ic | O00159 | + |  |
| 81 | NB5R3 | NADH-cytochrome b5 reductase 3 | P00387 | + | + |
| 82 | NDUS3 | NADH dehydrogenase [ubiquinone] iron-sulfur protein 3 | O75489 |  | + |
| 83 | NH2L1 | NHP2-like protein 1 | [P55769](http://www.uniprot.org/uniprot/P55769) | + | + |
| 84 | NHP2 | H/ACA ribonucleoprotein complex subunit 2 | [Q9NX24](http://www.uniprot.org/uniprot/Q9NX24) | + | + |
| 85 | NOL11 | Nucleolar protein 11 | [Q9H8H0](http://www.uniprot.org/uniprot/Q9H8H0) | + |  |
| 86 | NOLC1 | Nucleolar and coiled-body phosphoprotein 1 | [Q14978](http://www.uniprot.org/uniprot/Q14978) | + | + |
| 87 | NU155 | Nuclear pore complex protein Nup155 | O75694 | + |  |
| 88 | NUP53 | Nucleoporin NUP53 | [Q8NFH5](http://www.uniprot.org/uniprot/Q8NFH5) | + |  |
| 89 | PCNA | Proliferating cell nuclear antigen | P12004 | + |  |
| 90 | PDIA1 | Protein disulfide-isomerase | P07237 | + | + |
| 91 | PDIA3 | Protein disulfide-isomerase A3 | P30101 | + | + |
| 92 | PDIA6 | Protein disulfide-isomerase A6 | Q15084 | + | + |
| 93 | PESC | Pescadilio homolog | [O00541](http://www.uniprot.org/uniprot/O00541) | + | + |
| 94 | PGRC2 | Membrane-associated progesterone receptor component 2 | O15173 | + | + |
| 95 | PLST | Plastin-3 | [P13797](http://www.uniprot.org/uniprot/P13797) | + |  |
| 96 | PP1A | Serine/threonine-protein phosphatase PP1-alpha catalytic subunit | [P62136](http://www.uniprot.org/uniprot/P62136) | + |  |
| 97 | PRDX3 | Thioredoxin-dependent peroxide reductase, mitochondrial | P30048 | + | + |
| 98 | PRP6 | Pre-mRNA-processing factor 6 | [O94906](http://www.uniprot.org/uniprot/O94906) | + |  |
| 99 | PRP8 | Pre-mRNA-processing splicing factor 8 | [Q6P2Q9](http://www.uniprot.org/uniprot/Q6P2Q9) | + |  |
| 100 | PRS4 | 26S proteasome regulatory subunit 4 | [P62191](http://www.uniprot.org/uniprot/P62191) | + |  |
| 101 | PSMD1 | 26S proteasome non-ATPase regulatory subunit 1 | [Q99460](http://www.uniprot.org/uniprot/Q99460) | + | + |
| 102 | PSMD2 | 26S proteasome non-ATPase regulatory subunit 2 | Q13200 | + | + |
| 103 | PTBP1 | Polypyrimidine tract-binding protein 1 | [P26599](http://www.uniprot.org/uniprot/P26599) | + | + |
| 104 | PUF60 | Poly(U)-binding-splicing factor PUF60 | [Q9UHX1](http://www.uniprot.org/uniprot/Q9UHX1) | + | + |
| 105 | PUR9 | Bifunctional purine biosynthesis protein PURH | P31939 |  | + |
| 106 | RAB10 | Ras-related protein Rab-10 | P61026 | + | + |
| 107 | RAB14 | Ras-related protein Rab-14 | P61106 | + | + |
| 108 | RAB2A | Ras-related protein Rab-2A | P61019 | + | + |
| 109 | RAB5C | Ras-related protein Rab-5C | P51148 | + | + |
| 110 | RAB7A | Ras-related protein Rab-7a | P51149 | + | + |
| 111 | RALA | Ras-related protein Ral-A | [P11233](http://www.uniprot.org/uniprot/P11233) | + | + |
| 112 | RAP1B | Ras-related protein Rap-1b | P61224 | + | + |
| 113 | RB11A | Ras-related protein Rab-11A | P62491 |  | + |
| 114 | RBM14 | RNA-binding protein 14 | [Q96PK6](http://www.uniprot.org/uniprot/Q96PK6) | + | + |
| 115 | RBM3 | Putative RNA-binding protein 3 | [P98179](http://www.uniprot.org/uniprot/P98179) | + | + |
| 116 | RCC1 | Regulator of chromosomes condensation | [P18754](http://www.uniprot.org/uniprot/P18754) | + |  |
| 117 | RECQ1 | ATP-dependent DANN helicase Q1 | [P46063](http://www.uniprot.org/uniprot/P46063) | + |  |
| 118 | RFC4 | Replication factor C subunit 4 | [P35249](http://www.uniprot.org/uniprot/P35249) | + |  |
| 119 | RFC5 | Replication factor C subunit 5 | [P40937](http://www.uniprot.org/uniprot/P40937) | + |  |
| 120 | RL32 | 60S ribosomal protein L32 | [P62910](http://www.uniprot.org/uniprot/P62910) | + |  |
| 121 | RL7L | 60S ribosomal protein 7L-like 1 | [Q6DKI1](http://www.uniprot.org/uniprot/Q6DKI1) | + |  |
| 122 | RM12 | 39S ribosomal protein L12, mitochondrial | P52815 | + | + |
| 123 | RM13 | 39S ribosomal protein L13, mitochondrial | Q9BYD1 | + |  |
| 124 | RM22 | 39S ribosomal protein L22, mitochondrial | Q9NWU5 | + |  |
| 125 | RM43 | 39S ribosomal protein L43, mitochondrial | Q8N983 | + | + |
| 126 | RM48 | 39S ribosomal protein L48, mitochondrial | Q96GC5 | + |  |
| 127 | RPN2 | Dolichyl-diphosphooligosaccharide-protein glycosyltransferase subunit 2 | P04844 | + | + |
| 128 | RRP1 | Ribosomal RNA processing protein 1 homolog A | P56182 | + | + |
| 129 | RRP1B | Ribosomal RNA processing protein 1 homolog B | [Q14684](http://www.uniprot.org/uniprot/Q14684) | + | + |
| 130 | RS15 | 40S ribosomal protein S15a | [P62841](http://www.uniprot.org/uniprot/P62841) | + |  |
| 131 | RS24 | 40S ribosomal protein S24 | [P62847](http://www.uniprot.org/uniprot/P62847) | + |  |
| 132 | RTCB | tRNA-splicing ligase RtcB homolog | [Q9Y3I0](http://www.uniprot.org/uniprot/Q9Y3I0) | + | + |
| 133 | RU2B | U2 small nuclear ribonucleoprotein B'' | [P08579](http://www.uniprot.org/uniprot/P08579) | + |  |
| 134 | SAFB1 | Scaffold attachment factor B1 | [Q15424](http://www.uniprot.org/uniprot/Q15424) | + |  |
| 135 | SAP18 | Histone deacetylase complex subunit SAP18 | [O00422](http://www.uniprot.org/uniprot/O00422) | + | + |
| 136 | SAR1A | GTP-binding protein SAR1a | Q9NR31 | + |  |
| 137 | SC23A | Protein transport protein Sec23A | Q15436 | + |  |
| 138 | SC61B | Protein transport protein Sec61 subunit beta | [P60468](http://www.uniprot.org/uniprot/P60468) | + |  |
| 139 | SF3A3 | Splicing factor 3A subunit 3 | [Q12874](http://www.uniprot.org/uniprot/Q12874) | + | + |
| 140 | SLIRP | SRA stem-loop-interacting RNA-binding protein, mitochondrial | Q9GZT3 | + |  |
| 141 | SMC1A | Structural maintenance of chromosomes protein 1A | Q14683 | + | + |
| 142 | SMC3 | Structural maintenance of chromosomes protein 3 | Q9UQE7 | + | + |
| 143 | SNAA | Alpha-soluble NSF attachment protein | P54920 | + | + |
| 144 | SNR40 | U5 small nuclear ribonucleoprotein 40 kDa protein | [Q96DI7](http://www.uniprot.org/uniprot/Q96DI7) | + |  |
| 145 | SNRPA | U1 small nuclear ribonucleoprotein A | [P09012](http://www.uniprot.org/uniprot/P09012) | + | + |
| 146 | SNUT2 | U4/U6.U5 tri-snRNP-associated proetin 2 | [Q53GS9](http://www.uniprot.org/uniprot/Q53GS9) | + |  |
| 147 | SPTB2 | Spectrin beta chain, non-erythrocytic 1 | [Q01082](http://www.uniprot.org/uniprot/Q01082) | + |  |
| 148 | SRP72 | Signal recognition particle subunit SRP72 | O76094 | + |  |
| 149 | SRP14 | Signal recognition particle subunit 14 | [P37108](http://www.uniprot.org/uniprot/P37108) | + | + |
| 150 | SRPR | Signal recognition particle receptor subunit alpha | P08240 | + | + |
| 151 | SYLC | Leucine--tRNA ligase, cytoplasmic | [Q9P2J5](http://www.uniprot.org/uniprot/Q9P2J5) | + |  |
| 152 | SYMC | Methionine--tRNA ligase, cytoplasmic | [P56192](http://www.uniprot.org/uniprot/P56192) | + |  |
| 153 | SYMPK | Symplekin | [Q92797](http://www.uniprot.org/uniprot/Q92797) |  | + |
| 154 | SYNE1 | Nesprin 1 | [Q8NF91](http://www.uniprot.org/uniprot/Q8NF91) |  | + |
| 155 | SYQ | Glutamine--tRNA ligase | [P47897](http://www.uniprot.org/uniprot/P47897) | + |  |
| 156 | SYRC | Arginyl-tRNA synthetase, cytoplasmic | P54136 | + | + |
| 157 | SYVC | Valine--tRNA ligase | [P26640](http://www.uniprot.org/uniprot/P26640) | + |  |
| 158 | TBL3 | Transducin beta-like protein 3 | [Q12788](http://www.uniprot.org/uniprot/Q12788) | + |  |
| 159 | TCPE | T-complex protein 1 subunit epsilon | P48643 | + | + |
| 160 | TCPG | T-complex protein 1 subunit gamma | P49368 | + | + |
| 161 | TCPZ | T-complex protein 1 subunit zeta | P40227 | + | + |
| 162 | TDIF2 | Deoxynucleotidyltransferase terminal-interacting protein 2 | [Q5QJE6](http://www.uniprot.org/uniprot/Q5QJE6) | + |  |
| 163 | TERA | Transitional endoplasmic reticulum ATPase | P55072 | + | + |
| 164 | TFR1 | Transferrin receptor protein 1 | P02786 | + | + |
| 165 | THOC1 | THO complex subunit 1 | [Q96FV9](http://www.uniprot.org/uniprot/Q96FV9) |  | + |
| 166 | TIF1B | Transcription intermediary factor 1-beta | Q13263 | + | + |
| 167 | TMED9 | Transmembrane emp24 domain-containing protein 9 | Q9BVK6 | + | + |
| 168 | TMEDA | Transmembrane emp24 domain-containing protein 10 | P49755 | + | + |
| 169 | TMM43 | Transmembrane protein 43 | Q9BTV4 | + |  |
| 170 | TMOD3 | Tropomodulin-3 | [Q9NYL9](http://www.uniprot.org/uniprot/Q9NYL9) | + |  |
| 171 | TNPO1 | Transportin-1 | [Q92973](http://www.uniprot.org/uniprot/Q92973) | + |  |
| 172 | TOIP1 | Torsin-1A-interacting protein 1 | [Q5JTV8](http://www.uniprot.org/uniprot/Q5JTV8) | + |  |
| 173 | TOP2B | DNA topoisomerase 2-beta | Q02880 | + | + |
| 174 | TPM3 | Tropomyosin alpha-3 chain | [P06753](http://www.uniprot.org/uniprot/P06753) |  | + |
| 175 | TR150 | Thyroid hormone receptor-associated protein 3 | [Q9Y2W1](http://www.uniprot.org/uniprot/Q9Y2W1) | + |  |
| 176 | TRAP1 | Heat shock protein 75 kDa, mitochondrial | Q12931 |  | + |
| 177 | U520 | US small nuclear ribonucleoprotein 200 kDa helicase | [O75643](http://www.uniprot.org/uniprot/O75643) | + |  |
| 178 | UBA1 | Ubiquitin-like modifier-activating enzyme 1 | P22314 | + | + |
| 179 | UT14A | U3 small nucleolar RNA-associated protein 14 homolog A | [Q9BVJ6](http://www.uniprot.org/uniprot/Q9BVJ6) | + |  |
| 180 | VAPA | Vesicle-associated membrane protein A | Q9P0L0 | + | + |
| 181 | VIGLN | Vigilin | Q00341 | + | + |
| 182 | WDR43 | WD repeat-containing protein 43 | [Q15061](http://www.uniprot.org/uniprot/Q15061) | + |  |
| 183 | ZN207 | Zinc finger protein 207 | [O43670](http://www.uniprot.org/uniprot/O43670) | + |  |

**Table S3.** MS-identified proteins observed in *Legionella* containing vacuoles (LCV), *Salmonella*-modified membranes (SMM) and *Simkania*-containing vacuoles-ER-membranes (SnCV-ER).

| **#** | **Accession UniProtAC human** | **Protein description** | **Accession UniProtID human^1^** | **Accession UniProtID mouse^2^** | **Accession UniProtID dicti^3^** | **Detection HeLa SnCV** | **Detection THP1 SnCV** |
| --- | --- | --- | --- | --- | --- | --- | --- |
| 1 | 2AAA | Serine/threonine-protein phosphatase 2A 65 kDa | P30153 | Q76MZ3 |  | + | + |
| 2 | ACLY | ATP-citrate synthase | P53396 |  | Q54YA0 | + | + |
| 3 | ALDOA | Fructose-bisphosphate aldolase A | P04075 |  | Q86A67 | + | + |
| 4 | ARPC4 | Actin-related protein 2/3 complex subunit 4 | P59998 | P59999 | O96625 | + | + |
| 5 | C1TM | Monofunctional C1-tetrahydrofolate synthase, mitochondrial | Q6UB35 | Q3V3R1 |  | + |  |
| 6 | CAPZB | F-actin-capping protein subunit beta | P47756 | P47757 |  | + | + |
| 7 | DHB4 | Peroxisomal multifunctional enzyme type 2 | P51659 | P51660 | Q9NKW1 | + | + |
| 8 | DHX30 | Putative ATP-dependent RNA helicase DHX30 | Q7L2E3 | Q99PU8 |  | + |  |
| 9 | EF1B | Elongation factor 1-beta | P24534 | O70251 | Q9GRF8 | + | + |
| 10 | EF1G | Elongation factor 1-gamma | P26641 | Q9D8N0 |  | + | + |
| 11 | EF2 | Elongation factor 2 | P13639 | P58252 | P15112 | + | + |
| 12 | IMDH2 | Inosine-5'-monophosphate dehydrogenase 2 | P12268 | P24547 |  | + | + |
| 13 | MYH9 | Myosin-9 | P35579 | Q8VDD5 |  | + | + |
| 14 | PSMD2 | 26S proteasome non-ATPase regulatory subunit 2 | Q13200 | Q8VDM4 |  | + | + |
| 15 | PUR9 | Bifunctional purine biosynthesis protein PURH | P31939 |  | Q86L14 |  | + |
| 16 | RM12 | 39S ribosomal protein L12, mitochondrial | P52815 | Q9DB15 | Q86KA1 | + | + |
| 17 | RM13 | 39S ribosomal protein L13, mitochondrial | Q9BYD1 | Q9D1P0 |  | + |  |
| 18 | RM22 | 39S ribosomal protein L22, mitochondrial | Q9NWU5 | Q8BU88 |  | + |  |
| 19 | RM43 | 39S ribosomal protein L43, mitochondrial | Q8N983 | Q5RL20 |  | + |  |
| 20 | RM48 | 39S ribosomal protein L48, mitochondrial | Q96GC5 | Q8JZS9 |  | + |  |
| 21 | RRP1 | Ribosomal RNA processing protein 1 homolog A | P56182 | P56183 |  | + | + |
| 22 | SLIRP | SRA stem-loop-interacting RNA-binding protein, mitochondrial | Q9GZT3 |  | Q86I7L | + |  |
| 23 | SMC1A | Structural maintenance of chromosomes protein 1A | Q14683 | Q9CU62 |  | + | + |
| 24 | SMC3 | Structural maintenance of chromosomes protein 3 | Q9UQE7 | Q9CW03 |  | + | + |
| 25 | SRP72 | Signal recognition particle subunit SRP72 | O76094 | F8VQC1 |  | + |  |
| 26 | SYRC | Arginyl-tRNA synthetase, cytoplasmic | P54136 | Q9D0I9 | Q54JT6 | + | + |
| 27 | TCPE | T-complex protein 1 subunit epsilon | P48643 | P80316 |  | + | + |
| 28 | TCPG | T-complex protein 1 subunit gamma | P49368 | P80318 |  | + | + |
| 29 | TCPZ | T-complex protein 1 subunit zeta | P40227 | P80317 |  | + | + |
| 30 | TERA | Transitional endoplasmic reticulum ATPase | P55072 | Q01853 | P90532 | + | + |
| 31 | TIF1B | Transcription intermediary factor 1-beta | Q13263 | Q62318 |  | + | + |
| 32 | BAG2 | BAG family molecular chaperone regulator 2 | O95816 | Q91YN9 |  | + | + |
| 33 | ACTN1 | Alpha-actinin-1 | P12814 |  | P05095 | + | + |
| 34 | COR1C | Coronin-1C | Q9ULV4 |  | P27133 | + | + |
| 35 | DDX3X | ATP-dependent RNA helicase DDX3X | O00571 | Q62167 | Q54QS3 | + | + |
| 36 | MYO1C | Myosin-Ic | O00159 | Q9WTI7 |  | + |  |
| 37 | ANXA1 | Annexin A1 | P04083 | P10107 |  | + | + |
| 38 | AP2A1 | AP-2 complex subunit alpha-1 | O95782 | P17426 |  | + | + |
| 39 | AT5F1 | ATP synthase subunit b, mitochondrial | P24539 | Q9CQQ7 |  | + | + |
| 40 | ATD3A | ATPase family AAA domain-containing protein 3A | Q9NVI7 | Q925I1 |  | + |  |
| 41 | ATPG | ATP synthase subunit gamma, mitochondrial | P36542 | Q91VR2 | Q54DF1 | + | + |
| 42 | ATPO | ATP synthase subunit O, mitochondrial | P48047 | Q9DB20 | Q54RA8 | + | + |
| 43 | BAP31 | B-cell receptor-associated protein 31 | P51572 | Q61335 |  | + | + |
| 44 | CALX | Calnexin | P27824 | P35564 | Q55BA8 | + | + |
| 45 | COPA | Coatomer subunit alpha | P53621 | Q8CIE6 |  | + | + |
| 46 | COPG1 | Coatomer subunit gamma -1 | Q9Y678 | Q9QZE5 |  | + |  |
| 47 | EHD4 | EH domain-containing protein 4 | Q9H223 | Q9EQP2 |  |  | + |
| 48 | ENPL | Endoplasmin | P14625 | P08113 |  | + | + |
| 49 | HYOU1 | Hypoxia up-regulated protein 1 | Q9Y4L1 | Q9JKR6 | Q556U6 |  | + |
| 50 | IMMT | Mitochondrial inner membrane protein | Q16891 | Q8CAQ8 |  |  | + |
| 51 | LMNB2 | Lamin-B2 | Q03252 | P21619 |  | + |  |
| 52 | M2OM | Mitochondrial 2-oxoglutarate/malate carrier protein | Q02978 | Q9CR62 |  |  | + |
| 53 | MPCP | Phosphate carrier protein, mitochondrial | Q00325 | Q8VEM8 | Q54BF6 | + |  |
| 54 | MYH10 | Myosin-10 | P35580 |  | P08799 | + |  |
| 55 | NB5R3 | NADH-cytochrome b5 reductase 3 | P00387 | Q9DCN2 |  | + | + |
| 56 | NDUS3 | NADH dehydrogenase [ubiquinone] iron-sulfur protein 3 | O75489 | Q9DCT2 | P22237 |  | + |
| 57 | NU155 | Nuclear pore complex protein Nup155 | O75694 | Q99P88 |  | + |  |
| 58 | PCNA | Proliferating cell nuclear antigen | P12004 | P17918 |  | + |  |
| 59 | PDIA1 | Protein disulfide-isomerase | P07237 | P09103 |  | + | + |
| 60 | PDIA3 | Protein disulfide-isomerase A3 | P30101 | P27773 | Q54EN4 | + | + |
| 61 | PDIA6 | Protein disulfide-isomerase A6 | Q15084 |  | Q869Z0 | + | + |
| 62 | PRDX3 | Thioredoxin-dependent peroxide reductase, mitochondrial | P30048 | P20108 |  | + | + |
| 63 | RAB10 | Ras-related protein Rab-10 | P61026 | P61027 |  | + | + |
| 64 | RAB14 | Ras-related protein Rab-14 | P61106 | Q91V41 | P36410 | + | + |
| 65 | RAB2A | Ras-related protein Rab-2A | P61019 | P53994 | P36409 | + | + |
| 66 | RAB5C | Ras-related protein Rab-5C | P51148 | P35278 |  | + | + |
| 67 | RAB7A | Ras-related protein Rab-7a | P51149 | P51150 | P36411 | + | + |
| 68 | RAP1B | Ras-related protein Rap-1b | P61224 |  | P18613 | + | + |
| 69 | RB11A | Ras-related protein Rab-11A | P62491 | P62492 |  |  | + |
| 70 | RPN2 | Dolichyl-diphosphooligosaccharide-protein glycosyltransferase subunit 2 | P04844 | Q9DBG6 |  | + | + |
| 71 | SAR1A | GTP-binding protein SAR1a | Q9NR31 |  | Q559R0 | + |  |
| 72 | SNAA | Alpha-soluble NSF attachment protein | P54920 | Q9DB05 |  | + | + |
| 73 | SRPR | Signal recognition particle receptor subunit alpha | P08240 | Q9DBG7 | Q54ZR7 | + | + |
| 74 | TFR1 | Transferrin receptor protein 1 | P02786 | Q62351 |  | + | + |
| 75 | TMED9 | Transmembrane emp24 domain-containing protein 9 | Q9BVK6 | Q99KF1 | Q769F7 | + | + |
| 76 | TMEDA | Transmembrane emp24 domain-containing protein 10 | P49755 | Q9D1D4 | Q769F6 | + | + |
| 77 | TMM43 | Transmembrane protein 43 | Q9BTV4 | Q9DBS1 |  | + |  |
| 78 | TOP2B | DNA topoisomerase 2-beta | Q02880 |  | Q55BP5 | + | + |
| 79 | TRAP1 | Heat shock protein 75 kDa, mitochondrial | Q12931 | Q9CQN1 | Q86L04 |  |  |
| 80 | VAPA | Vesicle-associated membrane protein A | Q9P0L0 | Q9WV55 |  | + | + |
| 81 | VIGLN | Vigilin | Q00341 | Q8VDJ3 |  | + | + |
| 82 | 4F2 | 4F2 cell-surface antigen heavy chain | P08195 | P10852 |  | + | + |
| 83 | PGRC2 | Membrane-associated progesterone receptor component 2 | O15173 | Q80UU9 |  | + | + |
| 84 | K6PP | 6-phosphofructokinase type C | Q01813 | Q9WUA3 |  | + | + |
| 85 | UBA1 | Ubiquitin-like modifier-activating enzyme 1 | P22314 | Q02053 |  | + | + |

1,SMM proteins isolated from HeLa (data set: (1)); 2,LCV proteins isolated from RAW macrophages (data set: (2); 3,LCV proteins isolated from *Dictyostelium* strains (combined data sets: (2, 3)).

1. Vorwerk, S., Krieger, V., , J., Hensel, M., and Hansmeier N. (2014) Proteomes of host cells membranes modified by intracellular activities of *Salmonella enterica*. *Mol. Cell. Proteomics* 14(1), 81-92

2. Hoffmann, C., Finsel, I., Otto, A., Pfaffinger, G., Rothmeier, E., Hecker, M., Becher, D., and Hilbi, H. (2013) Functional analysis of novel Rab GTPases identified in the proteome of purified *Legionella*-containing vacuoles from macrophages. *Cell. Microbiol* 16, 1034-52

3. Shevchuk, O., Batzilla, C., Hagele, S., Kusch, H., Engelmann, S., Hecker, M., Haas, A., Heuner, K., Glockner, G., and Steinert, M. (2009) Proteomic analysis of *Legionella*-containing phagosomes isolated from *Dictyostelium*. *Int. J. Med. Microbiol* 299, 489-508

**Table S4.** MS-identified proteins observed in *Legionella* containing vacuoles (LCV), *Salmonella*-modified membranes (SMM) and *Chlamydia*-containing vacuoles (CCV).

| **#** | **Accession UniProtAC human** | **Protein description** | **Accession UniProtID human^1^** | **Accession UniProtID mouse^2^** | **Accession UniProtID dicti^3^** |
| --- | --- | --- | --- | --- | --- |
| 1 | ACLY | ATP-citrate synthase | P53396 |  | Q54YA0 |
| 2 | ALDOA | Fructose-bisphosphate aldolase A | P04075 |  | Q86A67 |
| 3 | ARPC4 | Actin-related protein 2/3 complex subunit 4 | P59998 | P59999 | O96625 |
| 4 | C1TM | Monofunctional C1-tetrahydrofolate synthase, mitochondrial | Q6UB35 | Q3V3R1 |  |
| 5 | CAPZB | F-actin-capping protein subunit beta | P47756 | P47757 |  |
| 6 | DHB4 | Peroxisomal multifunctional enzyme type 2 | P51659 | P51660 | Q9NKW1 |
| 7 | DHX30 | Putative ATP-dependent RNA helicase DHX30 | Q7L2E3 | Q99PU8 |  |
| 8 | EF1B | Elongation factor 1-beta | P24534 | O70251 | Q9GRF8 |
| 9 | EF1G | Elongation factor 1-gamma | P26641 | Q9D8N0 |  |
| 10 | EF2 | Elongation factor 2 | P13639 | P58252 | P15112 |
| 11 | IMDH2 | Inosine-5'-monophosphate dehydrogenase 2 | P12268 | P24547 |  |
| 12 | MYH9 | Myosin-9 | P35579 | Q8VDD5 |  |
| 13 | PSMD2 | 26S proteasome non-ATPase regulatory subunit 2 | Q13200 | Q8VDM4 |  |
| 14 | RM12 | 39S ribosomal protein L12, mitochondrial | P52815 | Q9DB15 | Q86KA1 |
| 15 | RM43 | 39S ribosomal protein L43, mitochondrial | Q8N983 | Q5RL20 |  |
| 16 | RM48 | 39S ribosomal protein L48, mitochondrial | Q96GC5 | Q8JZS9 |  |
| 17 | SLIRP | SRA stem-loop-interacting RNA-binding protein, mitochondrial | Q9GZT3 |  | Q86I7L |
| 18 | TCPE | T-complex protein 1 subunit epsilon | P48643 | P80316 |  |
| 19 | TCPG | T-complex protein 1 subunit gamma | P49368 | P80318 |  |
| 20 | TCPZ | T-complex protein 1 subunit zeta | P40227 | P80317 |  |
| 21 | TERA | Transitional endoplasmic reticulum ATPase | P55072 | Q01853 | P90532 |
| 22 | TIF1B | Transcription intermediary factor 1-beta | Q13263 | Q62318 |  |
| 23 | DDX3X | ATP-dependent RNA helicase DDX3X | O00571 | Q62167 | Q54QS3 |
| 24 | MYO1C | Myosin-Ic | O00159 | Q9WTI7 |  |
| 25 | ANXA1 | Annexin A1 | P04083 | P10107 |  |
| 26 | ATD3A | ATPase family AAA domain-containing protein 3A | Q9NVI7 | Q925I1 |  |
| 27 | ATPG | ATP synthase subunit gamma, mitochondrial | P36542 | Q91VR2 | Q54DF1 |
| 28 | ATPO | ATP synthase subunit O, mitochondrial | P48047 | Q9DB20 | Q54RA8 |
| 29 | CALX | Calnexin | P27824 | P35564 | Q55BA8 |
| 30 | COPG1 | Coatomer subunit gamma -1 | Q9Y678 | Q9QZE5 |  |
| 31 | ENPL | Endoplasmin | P14625 | P08113 |  |
| 32 | HYOU1 | Hypoxia up-regulated protein 1 | Q9Y4L1 | Q9JKR6 | Q556U6 |
| 33 | IMMT | Mitochondrial inner membrane protein | Q16891 | Q8CAQ8 |  |
| 34 | M2OM | Mitochondrial 2-oxoglutarate/malate carrier protein | Q02978 | Q9CR62 |  |
| 35 | MPCP | Phosphate carrier protein, mitochondrial | Q00325 | Q8VEM8 | Q54BF6 |
| 36 | NB5R3 | NADH-cytochrome b5 reductase 3 | P00387 | Q9DCN2 |  |
| 37 | NDUS3 | NADH dehydrogenase [ubiquinone] iron-sulfur protein 3 | O75489 | Q9DCT2 | P22237 |
| 38 | NU155 | Nuclear pore complex protein Nup155 | O75694 | Q99P88 |  |
| 39 | PDIA1 | Protein disulfide-isomerase | P07237 | P09103 |  |
| 40 | PDIA3 | Protein disulfide-isomerase A3 | P30101 | P27773 | Q54EN4 |
| 41 | PDIA6 | Protein disulfide-isomerase A6 | Q15084 |  | Q869Z0 |
| 42 | PRDX3 | Thioredoxin-dependent peroxide reductase, mitochondrial | P30048 | P20108 |  |
| 43 | RAB10 | Ras-related protein Rab-10 | P61026 | P61027 |  |
| 44 | RAB14 | Ras-related protein Rab-14 | P61106 | Q91V41 | P36410 |
| 45 | RAB2A | Ras-related protein Rab-2A | P61019 | P53994 | P36409 |
| 46 | RAB5C | Ras-related protein Rab-5C | P51148 | P35278 |  |
| 47 | RAB7A | Ras-related protein Rab-7a | P51149 | P51150 | P36411 |
| 48 | RAP1B | Ras-related protein Rap-1b | P61224 |  | P18613 |
| 49 | RPN2 | Dolichyl-diphosphooligosaccharide-protein glycosyltransferase subunit 2 | P04844 | Q9DBG6 |  |
| 50 | SNAA | Alpha-soluble NSF attachment protein | P54920 | Q9DB05 |  |
| 51 | TFR1 | Transferrin receptor protein 1 | P02786 | Q62351 |  |
| 52 | TRAP1 | Heat shock protein 75 kDa, mitochondrial | Q12931 | Q9CQN1 | Q86L04 |
| 53 | VIGLN | Vigilin | Q00341 | Q8VDJ3 |  |
| 54 | 4F2 | 4F2 cell-surface antigen heavy chain | P08195 | P10852 |  |
| 55 | PGRC2 | Membrane-associated progesterone receptor component 2 | O15173 | Q80UU9 |  |
| 56 | K6PP | 6-phosphofructokinase type C | Q01813 | Q9WUA3 |  |
| 57 | UBA1 | Ubiquitin-like modifier-activating enzyme 1 | P22314 | Q02053 |  |

1,SMM proteins isolated from HeLa (data set: (1)); 2,LCV proteins isolated from RAW macrophages (data set: (2); 3,LCV proteins isolated from *Dictyostelium* strains (combined data sets: (2, 3)).

1. Vorwerk, S., Krieger, V., , J., Hensel, M., and Hansmeier N. (2014) Proteomes of host cells membranes modified by intracellular activities of *Salmonella enterica*. *Mol. Cell. Proteomics* 14(1), 81-92

2. Hoffmann, C., Finsel, I., Otto, A., Pfaffinger, G., Rothmeier, E., Hecker, M., Becher, D., and Hilbi, H. (2013) Functional analysis of novel Rab GTPases identified in the proteome of purified *Legionella*-containing vacuoles from macrophages. *Cell. Microbiol* 16, 1034-52

3. Shevchuk, O., Batzilla, C., Hagele, S., Kusch, H., Engelmann, S., Hecker, M., Haas, A., Heuner, K., Glockner, G., and Steinert, M. (2009) Proteomic analysis of *Legionella*-containing phagosomes isolated from *Dictyostelium*. *Int. J. Med. Microbiol* 299, 489-508
